# Increased thermal stability of FGF10 leads to ectopic signaling during development

**DOI:** 10.1007/s00018-025-05681-1

**Published:** 2025-04-21

**Authors:** Aleksandra A. Czyrek, Karolina Baran, Eva Hruba, Aneta Horackova, Veronika Bosakova, Julia Chudzian, Bohumil Fafilek, Veronika Laskova, Veronika Stepankova, David Bednar, Kelly Karl, Petr Kasparek, Michaela Bosakova, Michal Killinger, Tereza Szotkowska, Jan Prochazka, Jennifer T. Zieba, Gustavo Rico-Llanos, Jan Fric, Stefan Hadzic, Edma Loku, Magdalena Wujak, Katerina Svozilova, Michaela Stroblova, Radislav Sedlacek, Kalina Hristova, Deborah Krakow, Jan Kubovciak, Mathys Delattre, Rafal Bartoszewski, Marcela Buchtova, Daniel Krowarsch, Radka Chaloupkova, Malgorzata Zakrzewska, Pavel Krejci

**Affiliations:** 1 Department of Biology, Faculty of Medicine, Masaryk University, Brno, 62500 Czech Republic; 2 International Clinical Research Center, St. Anne’s University Hospital, Brno, 65691 Czech Republic; 3 Department of Protein Engineering, Faculty of Biotechnology, University of Wroclaw, Wroclaw, 50-383 Poland; 4 Institute of Animal Physiology and Genetics, Czech Academy of Sciences, Brno, 60200 Czech Republic; 5 Department of Experimental Biology, Faculty of Science, Masaryk University, Brno, 62500 Czech Republic; 6 Enantis Ltd, Brno, 62500 Czech Republic; 7 Loschmidt Laboratories, Department of Experimental Biology and RECETOX, Faculty of Science, Masaryk University, Brno, 62500 Czech Republic; 8 Department of Materials Science and Engineering, Institute for NanoBioTechnology, and Program in Molecular Biophysics, Johns Hopkins University, Baltimore, MD 21218 USA; 9 Czech Center for Phenogenomics, Institute of Molecular Genetics of the Czech Academy of Sciences, Vestec, 25250 Czech Republic; 10 Department of Orthopaedic Surgery, Human Genetics, and Obstetrics and Gynecology, University of California Los Angeles, California Los Angeles, CA 90095 USA; 11 Institute of Hematology and Blood Transfusion, Prague, 12800 Czech Republic; 12 Excellence Cluster Cardio-Pulmonary Institute (CPI), Universities of Giessen and Marburg Lung Center (UGMLC), Member of the German Center for Lung Research (DZL), Justus Liebig University, 35392 Giessen, Germany; 13 Department of Medicinal Chemistry, Collegium Medicum in Bydgoszcz, Faculty of Pharmacy, Nicolaus Copernicus University in Torun, Bydgoszcz, 85-089 Poland; 14 Laboratory of Genomics and Bioinformatics, Institute of Molecular Genetics of the Czech Academy of Sciences, Prague, 14200 Czech Republic; 15 Department of Biophysics, Faculty of Biotechnology, University of Wroclaw, Wroclaw, 50-383 Poland

**Keywords:** Fibroblast growth factor, FGF10, Morphogen, Stability, Development, Lung

## Abstract

Fibroblast growth factors (FGFs) control organ morphogenesis during development as well as tissue homeostasis and repair in the adult organism. Despite their importance, many mechanisms that regulate FGF function are still poorly understood. Interestingly, the thermodynamic stability of 22 mammalian FGFs varies widely, with some FGFs remaining stable at body temperature for more than 24 h, while others lose their activity within minutes. How thermodynamic stability contributes to the function of FGFs during development remains unknown. Here we show that FGF10, an important limb and lung morphogen, exists as an intrinsically unstable protein that is prone to unfolding and is rapidly inactivated at 37 °C. Using rationally driven directed mutagenesis, we have developed several highly stable (STAB) FGF10 variants with a melting temperature of over 19 °C more than that of wildtype FGF10. In cellular assays in vitro, the FGF10-STABs did not differ from wildtype FGF10 in terms of binding to FGF receptors, activation of downstream FGF receptor signaling in cells, and induction of gene expression. In mouse embryonal lung explants, FGF10-STABs, but not wildtype FGF10, suppressed branching, resulting in increased alveolarization and expansion of epithelial tissue. Similarly, FGF10-STAB1, but not FGF10 wildtype, inhibited the growth of mouse embryonic tibias and markedly altered limb morphogenesis when implanted into chicken limb buds, collectively demonstrating that thermal instability should be considered an important regulator of FGF function that prevents ectopic signaling. Furthermore, we show enhanced differentiation of human iPSC-derived lung organoids and improved regeneration in ex vivo lung injury models mediated by FGF10-STABs, suggesting an application in cell therapy.

**Supplementary Information:**

The online version contains supplementary material available at 10.1007/s00018-025-05681-1.

## Introduction

The fibroblast growth factor (FGF) family consists of 22 proteins that function as morphogens, growth factors and metabolic hormones [1]. FGFs regulate many developmental processes in mammals, such as blastocyst formation (FGF4), gastrulation (FGF8), epithelial-mesenchymal interactions necessary for the development of epithelial (FGF10) or mesenchymal (FGF9, FGF18) components of multiple organs, heart and brain morphogenesis (FGF15, FGF16, FGF17), and others [2–7].

In their paracrine signaling, FGFs must remain stable in the extracellular space so that they can reach, bind and activate the FGF receptors (FGFRs) on the surface of the recipient cells. Several lines of evidence suggest that the low intrinsic stability of some FGF proteins impairs their function. At physiological temperatures, about 50% of FGF1 exists in an unfolded state that cannot activate FGFR signaling and is susceptible to proteolytic degradation [8]. The thermal stability of FGF1 can be improved by mutagenesis. Introduction of Q40P, S47I and H93G substitutions improved the thermal stability of FGF1 by more than 21 °C, ensured the correct conformation at physiological temperature and significantly improved FGF1’s biological activity [9, 10]. Thermal instability is not limited to FGF1, as FGF3, FGF4, FGF6, FGF8, FGF10, FGF20 and FGF22 were also found to be unstable and rapidly degraded in cell culture media [11, 12]. Even minor perturbations in FGF stability can lead to a significant change in its ability to activate FGFR signaling. For example, a decrease in thermal stability by only 3.5 °C and 4.6 °C resulted in a complete loss of biological activity of R109A/K110A and L140A mutants of FGF2 [12]. The binding of heparin significantly increased both the stability and biological activity of unstable FGFs.

Most experimental work addressing thermal stability has been performed with FGF1, which, however, does not play a significant role in development [13]. Therefore, the relationship between the thermal stability of FGFs and their function in mammalian development remains unknown. Interestingly, FGF10 appears to be the least stable FGF, with a half-life of less than 30 min in tissue culture media [12]. In this study, we investigated how thermal stability regulates FGF10 function. We developed stabilized FGF10 variants (FGF10-STABs) with melting temperatures increased by more than 19 °C compared to wildtype FGF10. FGF10-STABs exhibited normal binding and activation of FGFRs but induced morphological defects in embryonic lung and limb explant cultures due to ectopic signaling, suggesting that thermal stability plays a crucial role in regulating FGF function during morphogenesis.

## Materials and methods

### Cell culture, migration and proliferation assays, Western blot, KroxDs reporter assay and qPCR

RCS, MCF7 and 293T cells were propagated in DMEM media supplemented with 10% FBS and antibiotics (Invitrogen); 4MBr-5 cells were propagated in F12K medium (ATCC). Cell migration was measured using the IncuCyte Cell Migration Assay (Essen BioScience). 45,000 MCF7 cells/well were seeded on poly-D-lysine-coated 96-well plates, scratched with the IncuCyte WoundMaker and stimulated with FGF10 in the presence of 0.5% FBS for 48 h. Wound images were taken automatically every 2 h. For proliferation assays, 4MBr-5 cells were seeded in 24-well plates and treated with FGF10 (20 ng/ml) every other day for up to seven days. Recombinant FGF1 and FGF2 were obtained from Biotechne. *Fgfr1-4 null* cells were generated from RCS cells in which the endogenous *Fgfr* genes were inactivated by CRISPR/Cas9. The RCS cells expressing a single FGFR variant were generated by stable transfection of *Fgfr1-4 null* cells with vectors containing the single V5-tagged full-length human FGFR1-4 [14]. Stable integration was achieved by PiggyBac transposase, and low FGFR expression was ensured by an attenuated CMV promoter. KroxDs reporter (pKrox24(MapERK)d1EGFP; Addgene#214912) was developed by replacing DsRED in pKrox24(MapERK) DsRed reporter (Addgene#200114) [14] with destabilized d1EGFP complemented by the 3’-UTR of the mouse *Egr1* gene. The 12XCSL-d1EGFP vector was a gift from Urban Lendahl (Addgene#47684) [15]. KroxDs was stably integrated into MCF7 cells using PiggyBac transposase; transactivation was determined by automated microscopy (Nikon BioStation). For western blot, cells were harvested directly into Laemmli sample buffer; lysates were resolved by SDS-PAGE, transferred to a PVDF membrane and visualized by chemiluminescence (Thermo Fisher Scientific). Western blot signal was quantified using ImageJ software (http://imagej.nih.gov/ij/). Table S1 lists all used antibodies. For qPCR, total RNA was purified from whole embryonic explants using the Mini RNeasy Kit (Qiagen). The RNA was transcribed into cDNA using the gb Elite Reverse Transcription Kit (Generi Biotech). Fluorophore-labeled TaqMan probes were obtained from Thermo Fisher Scientific: *Cdh1* (Mm01247357_m1), *Sfptb* (Mm00455678_m1), *Sox2* (Mm03053810_s1), *Spry2* (Mm00442344_m1) and *Spry4* (Mm00442345_m1). Gene expression levels were calculated using the ∆∆Ct method with normalization against *Actb*. For gene expression analysis in lung organoids, the RNA was isolated by TRI Reagent^®^ (Merck) and column separation using RNeasy Plus Micro Kit with gDNA elimination (Qiagen) according to the manufacturer’s protocol. Gene expression was analyzed using qPCR TaqMan probes including *Cdh1* (Hs01023895_m1), *Sox9* (Hs00165814_m1), *Spry2* (Hs01921749_s1), and *Hprt1* (Hs02800695_m1) and TaqManTM Gene expression Master Mix (Thermo Fisher Scientific) according to the manufacturer’s protocol. The StepOne™ Real-Time PCR System (Applied Biosystems) was used for the qPCR analysis.

### RNA sequencing and differential expression analysis

For RNA-seq, the MCF7 cells were serum starved for two days and stimulated with 20 ng/ml FGF10 for 8 h, followed by total RNA isolation with the use of RNeasy Plus Kits for RNA Isolation with gDNA Eliminator columns (Qiagen). The quantity and quality of isolated RNA were measured using NanoDrop ND-2000 (NanoDrop Technologies) and analyzed by Agilent 2100 Bioanalyser (Agilent Technologies). Isolated RNA was processed using KAPA mRNA Hyperprep Kit (Roche, RN 08098123702) according to manufacturer’s instructions. Libraries were sequenced on the Illumina NextSeq 2000 instrument using P2 100 flowcell with 122 bp single-end read length. Data was processed using nf-core/rnaseq v3.17.0 [16] of the nf-core collection of workflows [17], utilizing reproducible software environments from the Bioconda [18] and Biocontainers [19] projects. Individual steps included removing sequencing adaptors and low-quality reads with Trim Galore v0.6.10 (http://www.bioinformatics.babraham.ac.uk/projects/trim_galore/) and cutadapt [20] v4.9, mapping to reference genome GRCh38 [21] (Ensembl annotation version 113) with STAR [22] v2.7.11b and quantifying expression on gene level with Salmon [23] v1.10.3. Per gene quantified fragment counts served as input for differential expression analysis using negative binomial GLM fitting and Wald test in DESeq2 [24] R Bioconductor package. Only genes with raw quantified expression higher than 10 across all samples were accounted for the testing. We supplied an experimental model assuming sample group as the main effect. Genes exhibiting absolute log_2_ fold change value of 1 or greater and statistical significance (adjusted *p*-value < 0.05) between compared groups of samples were considered as differentially expressed.

### FGF10-STAB1, FGF10-STAB2 and FGF10-STAB3 design, expression and purification

For FGF10-STAB1, the structure of the FGF10:FGFR2b complex (PDB:1NUN) [25] was used to predict the stabilizing substitutions in FGF10 by an energy-based approach described in the FireProt strategy [26]. The stability effects of all possible single-point mutations were estimated by the force field calculations using BuildModel module of FoldX [27] and ddg monomer module of Rosetta [28], both with default settings [29]. ΔΔG free energies were collected across all 20 mutations at a given position. Evolutionary conservation was estimated using phylogenetic analysis of homologous sequences. Mutations with ΔΔG<-1.0 kcal/mol and conservation ≤ 7 were selected for further analysis. Residues located in known FGFR and heparin binding sites were excluded. For evolution-based prediction of stabilizing substitutions, multiple-sequence alignment of human FGF10 (UniProt O15520) with related proteins was performed using PSI-BLAST. Sequences were clustered using CLANS [30] and aligned using MUSCLE [31]. The resulting alignment was used for back-to-consensus analysis using the consensus cut-off of 0.5, meaning that a given residue must be present at a given position in at least 50% of all analyzed sequences to be assigned as the consensus residue. Mutations identified by consensus have to also pass cut-off of ΔΔG < 0.5 kcal/mol. Selected single-point substitutions were combined in silico to form all possible two- and multiple-point mutants, and their energies were estimated using force field calculations. Mutants were designed using protocol 16, which accounts for backbone flexibility in the ddg monomer module of the Rosetta software [28]. Human FGF10 (40–208) cDNA was commercially synthesized (Thermo Fisher Scientific) and mutagenesis was performed by whole plasmid PCR using the pET28b-His-thrombin::*fgf10* vector as a template. Recombinant FGF10 single and multiple-point variants were expressed in *E. coli* BL21(DE3) and purified by nickel affinity chromatography [29]. FGF10-STAB2 and -STAB3 were developed by template-based thermostability engineering using a consensus approach [32] and rational 3D structure-based engineering [9]. The Pham database was used as a source of homologous FGF sequences [33], and a multiple sequence alignment was generated using Clustal W software. Residues involved in FGFR binding were identified using LigPlot software [34] based on the FGF10:FGFR2b complex (PDB: 1NUN) [25]. Heparin-binding residues were predicted based on the superposition of the FGF1:FGFR2c:heparin (PDB:1E0O) [35] and FGF2:FGFR1c:heparin (PDB:1FQ9) [36] complexes with the FGF10:FGFR2b structure. The FGF10 structure was analyzed using PROCHECK software [37] to determine the exact location of the secondary structure elements in the polypeptide chain. We estimated the potential improvement of secondary structure using the β-sheet and β-turn amino acid potentials for preselected mutations. In addition, analysis of allowed side chain rotamers (Swiss-PdbViewer), potential effects on the nearby hydrogen bond network and changes in solvent exposure were considered in the development of stable mutants. Recombinant single variants were obtained and characterized. Subsequently, the variants that increased thermal stability the most were analyzed in combination with others and multiple-point mutants were developed. FGF10 variant constructs (based on the wildtype 38–208 aa sequence) in the pET-3d vector (GeneUniversal) were used to produce recombinant proteins. The recombinant proteins were expressed in *E. coli* BL21(DE3)pLysS cultured in LB medium containing 0.01% ampicillin and 0.003% chloramphenicol to OD=0.8. Protein expression was induced by 1 mM IPTG (Iris Biotech) and bacteria were cultured overnight at 25 °C, harvested by centrifugation and sonicated in the buffer containing 50 mM Tris, 150 mM NaCl, 1 mM EDTA, 1 mM DTT, 1 mM PMSF, EDTA-free protease inhibitor cocktail (Roche) (pH 8.0). The supernatant was loaded onto HiTrap Heparin HP column (Cytiva) equilibrated with PBS (pH 7.4). The column was washed with 10 volumes of PBS and FGF10 variants were eluted with a linear gradient of NaCl (0.4–1.5 M) in PBS (pH 7.4).

### Biophysical characterization of FGF10-STABs, trypsin assay and biolayer interferometry (BLI)

The thermal stability of FGF10-STAB1 was determined by differential scanning fluorimetry (DSF), differential scanning calorimetry (DSC) and circular dichroism (CD) spectroscopy. Thermal unfolding of 0.2 mg/ml or 1.0 mg/ml protein solutions in 20 mM phosphate buffer pH 7.5 with 750 mM sodium chloride was determined by monitoring intrinsic fluorescence at 330/350 nm, the changes in heat capacity and ellipticity chances at 227 nm using the Prometheus DSF system (NanoTemper Technologies), the VP capillary DSC system (GE Healthcare) and the Chirascan spectropolarimeter (Applied Photophysics), respectively, at temperature range 25–90 °C and 1 °C/min heating rate. The melting temperature (T_m_) was evaluated as the midpoint of the normalized thermal fluorescence transitions, the maximum of the apparent heat capacity peaks and the midpoint of the normalized thermal ellipticity transitions. For FGF10-STAB2 and FGF10-STAB3, CD measurements were performed using a Jasco J-71,500 spectropolarimeter (JASCO). Thermal denaturation was monitored by changes in ellipticity at 228 nm with a slit width of 2 nm and a response time of 8s. Proteins at a concentration of 0.5 µM in phosphate buffer (25 mM H_3_PO_4_, pH 7.3) were heated at a constant rate of 1 °C/min. Data were analyzed assuming a two-state denaturation process using PeakFit (Systat software). The structural integrity of FGF10-STAB during incubation at 37 °C was monitored by CD spectroscopy. Changes in the secondary structure of wildtype FGF10 and FGF10-STAB1 at a concentration of 0.2 mg/ml in 20 mM phosphate buffer (pH 7.5) containing 750 mM NaCl were monitored by monitoring the ellipticity over the wavelength range of 200–260 nm at 37 °C. Data were recorded at 5 min intervals with a bandwidth of 1 nm. The recorded denaturation curves (single exponential function) of the tested FGF10 variants were globally fitted to exponential decay curves using Origin2019b software (OriginLab). The half-life of FGF10 secondary structure was defined as the time required to reduce the initial value of ellipticity, as a measure of the secondary structure of the protein, to half of the original value. Half-life was determined from the collected data as the decay constant (τ) using the following equation: t_1/2_ = ln (2)/λ = τ ln (2), where λ is the exponential decay constant. The t_1/2_ constant was determined from data collected at 227 nm, where the CD spectra of both FGF10 protein variants showed the ellipticity maxima. For all FGF10-STABs, the proteolytic degradation assay was performed at 37 °C in 100 mM Tris-HCl, 20 mM calcium chloride, pH 8.3, where the molar ratio of trypsin:FGF10 was 1:50. After the indicated times, the reaction was stopped with Laemmli sample buffer and the products were analyzed by SDS-PAGE. For biolayer interferometry (BLI) measurements, the fully glycosylated extracellular domains of FGFR1-3b fused to the Fc domain of IgG1 were purified similarly as described for FGFR1-3c isoforms [38]. The BLI experiments were performed on Octet K2 (Sartorius AG). The FGFR2b-Fc was immobilized on a protein A biosensor and incubated with FGF10 variants diluted in PBS at the concentration range from 31.25 to 250 nM. Association and dissociation were monitored for 300s each. The data obtained were analyzed using ForteBio Data Analysis 11.0 software. The equilibrium dissociation constants (K_D_) were calculated from fitted saturation binding curves.

### Fluorescence intensity fluctuations (FIF)

293T cells were seeded on collagen coated petri dishes and allowed to grow for 24 h before transfection. At ~ 70% confluency, the cells were transfected with 2 µg of DNA of ECTM-FGFR2b-eYFP using FuGENE HD (Promega). The membranes of transfected cells were imaged on a Leica SP8 confocal microscope using a photon counting detector. eYFP was excited using a 488 nm diode laser at 1% to avoid photobleaching, and a scanning speed of 20 Hz was used. Only one scan was performed. A region of interest (ROI) in the plasma membrane of each cell was manually outlined and selected for analysis. Each ROI was divided into segments of 15 × 15 (225 pixels) as described [39, 40]. Histograms of pixel intensities were constructed for each segment, and brightness values, $\:{{\upepsilon\:}}_{seg,\:\:}$ was calculated using the intensity and variance values for this segment.

$${\varepsilon _{seg}} = {1 \over \gamma }\left( {{{\sigma _{seg}^2} \over {{I_{seg}}}} - 1} \right)$$

A shape factor γ of 0.5 was included into the above equation as described, accounting for the beam intensity shape and orientation direction with respect to the beam direction [39]. Brightness values were binned and histogrammed for all the segments of at least 100 analyzed cells. Normalized frequency counts were plotted against brightness values and compared to a monomer control curve (LAT).

### Lung explant cultures, whole-mount immunohistochemistry and limb bud implantation

For explant cultures, pseudoglandular stage lungs with trachea were isolated from E11.25 mouse embryos (ICR line, Masaryk University), placed on a porous PET membrane (Corning) and kept floating in an IVF culture dish (SPL LifeSciences) containing 500 µl DMEM (Gibco) with 0.1% FBS and antibiotics. The medium supplemented with FGF10 was used throughout the culture period of 0–72 h and changed daily or twice a day. Tibiae harvested from E18.0 mouse embryos were placed on a porous PET membrane (Corning) over a metal mesh and cultured for 8 days in F12/DMEM supplemented with 10% FBS, ascorbic acid (50 µg/ml), 10 mM β-glycerol phosphate, 1% L-glutamine and antibiotics. The medium was changed daily. Tibial length was measured on day 0 and day 8 using Axio Vision (Zeiss). Lung explants were fixed in 4% PFA, permeabilized in PBS/0.5% Triton (PBST), blocked with 1% BSA and 10% FBS in PBST and incubated with primary antibodies for 60 h at 4ºC. After incubation, the explants were washed in PBST and incubated for 60 h with secondary antibodies in blocking solution. During incubation with the secondary antibodies, Draq5 was added overnight to stain the nuclei. Explants were washed in PBS, mounted in Fluoroshield (Sigma-Aldrich) in Nunc™ Glass Bottom Dishes (Thermo Fisher Scientific) and scanned with a Leica SP8 confocal microscope. Table S1 lists antibodies used for immunohistochemistry. Embryonic lung size, alveolar area and surface were quantified using ImageJ software (Fig. S10). Chicken embryos were obtained from INTEGRA (Zabcice, Czech Republic). FGF10 (1 mg/ml) was soaked for 1 h on ice in Affi-Gel Blue Beads (BioRad) with a diameter of 200 μm. The FGF10 beads were implanted into the right forelimb buds at stage HH19-21, while the left forelimbs were implanted with beads containing only PBS. The embryos were incubated until HH38. The wings were removed, skinned and fixed in 100% ethanol/96 h and acetone/96 h and stained in 5% acetic acid, 0.015% alcian blue and 0.005% alizarin red in 70% ethanol for 7 days. The forelimbs were cleared in 20% glycerol in 2% KOH for 1 month. All animal experiments were performed in accordance with the guidelines of the Institutional Animal Care and Use Committee at the Institute of Animal Physiology and Genetics ASCR and Masaryk University.

### Induced pluripotent stem cells (iPSC) culture and lung organoids (LOs) differentiation

Human induced pluripotent stem cells (hiPSCs) (WiCell, DF19-9-7T) [41] were cultivated on Cultrex Stem Cell Qualified Reduced Growth Factor Basement Membrane Extract (R&D Systems) - coated tissue culture dishes and maintained in mTeSR Plus medium (StemCell Technologies) with penicillin and streptomycin (500 U/ml) in the incubator at 37 °C, 5% CO_2_ and 95% humidity. Lung organoids (LO) were differentiated from iPSCs following an adapted protocol previously published [42–44]. In brief, iPSCs were differentiated into definitive endoderm using the three-step protocol. On the first day, the medium was changed and RPMI 1640 (Gibco) supplemented with Activin A (100 ng/ml, R&D Systems) was added. On the second day, the medium was changed for fresh RPMI 1640 supplemented with Activin A (100 ng/ml) and 0.2% HyClone-defined FBS (GE Healthcare). On the third and fourth days, the medium was replaced with fresh RPMI 1640 supplemented with activin A (100 ng/ml) and 2% FBS. After four days of differentiation, the endodermal layer was induced to foregut differentiation with Advanced DMEM F12 (Gibco) supplemented with N2, B27 and GlutaMAX supplements (Thermo), penicillin and streptomycin (500 U/ml), noggin (250 ng/ml; R&D Systems), SB431542 (10 µM; STEMCELL), SAG (1 µM; Tocris), FGF4 (500 ng/ml; R&D Systems) and CHIR-99021 (2 µM; STEMCELL) (basic LO media). After 4–5 days, 3D spheroids were formed. The spheroids were collected, embedded in Cultrex RGF Basement Membrane Extract, Type 2 (R&D Systems) drops, and fed with complete LO medium (basic LO medium supplemented with 1% HyClone-defined FBS and FGF10 (500 ng/ml). The medium was changed twice a week and LOs were used for the experiments after 50 days in the culture. For immunofluorescence analysis, LOs were washed with PBS and fixed with 4% paraformaldehyde (PFA) for 20 min at room temperature. LOs were permeabilized with 0.5% Triton X-100 in PBS and then washed three times with IF buffer (PBS with 0.2% Triton X-100 + 0.05% Tween-20). Samples were blocked with 2.5% bovine serum albumin (BSA) in IF buffer. The LOs were incubated with anti-human E-cadherin, and anti-SOX9 (Abcam) antibodies (Table S1) in IF buffer + 1% BSA at 4 °C overnight. The LOs were washed with IF buffer three times and incubated with secondary anti-rabbit (Alexa Fluor™ 647) and anti-rat antibodies (Alexa Fluor™ 488) in IF buffer + 1% BSA for one hour at room temperature. Images were acquired with Zeiss LSM 780 confocal microscope at 20x magnification. The E-cadherin^+^ area and automated cell count of SOX9^+^ cells was done using ImageJ software. The analysis was performed for at least two technical replicates (two acquired pictures from different parts of the same organoid) and three biological replicates (three independent organoids).

### Ex vivo early fibrosis and injury model in mouse precision-cut lung slices (PCLS) experiments

PCLS were generated from C57BL/6J mice (Charles River Laboratories International) [45, 46]. Lung lobes were isolated and cut transversely at 150 μm using an automated vibratome (Leica Microsystems). Mouse PCLS were placed in a 24-well plate in ice-cold M199 medium (Gibco) supplemented with 1x insulin/transferrin/selenium (Gibco), 1 µg/ml vitamin C, 0.1 µg/ml vitamin, and 0.1 µg/ml hydrocortisone (Merck Millipore). The medium was supplemented with 1% (v/v) penicillin-streptomycin (Gibco) and 100 µg/ml normocin (InvivoGen). For the experiments, the medium was changed for the M199 medium containing the above supplements and serum replacement 1% panexin (PAN-Biotech). To recapitulate pro-fibrotic events described in pulmonary fibrosis, the fibrotic cocktail (FC) was prepared as previously reported, with minor modifications [47]. FC was prepared in the complete M199 medium and contained 5 ng/ml recombinant TGFβ1, 10 ng/ml PDGFAB, 10 ng/ml TNFα (R&D Systems), and 5 µM 1-oleoyl lysophosphatidic acid (LPA) (Cayman Chemical). The mouse PCLS were incubated with FC or vehicle for 24 and 72 h for imaging/proliferation and cytotoxicity assessment, respectively. To mimic the emphysema-like changes in mouse lungs ex vivo, PCLS were treated with elastase [48]. Briefly, PCLS were incubated with 2.5 µg/ml elastase (Merck Millipore) or vehicle (saline) for 24 h. FGF10 variants were used at a concentration of 250 ng/ml for ex vivo PCLS treatment [49]. Cytotoxicity was assessed using the CyQUANT™ LDH cytotoxicity assay (Invitrogen) [46]. Absorbance at 490 nm and 680 nm was measured using a Spark multimode microplate reader (Tecan Life Sciences). The measured cytotoxicity values were then related to PCLS weight to exclude the effects of size differences. The metabolic activity assessment of PCLS was performed by alamarBlue assay (Invitrogen). The fluorescent signal was determined using a Spark multimode microplate reader. For the immunostaining, PCLS were washed with PBS, fixed with 4% PFA (ThermoFisher) for 20 min and permeabilized with 0.1% Triton-X in PBS. Afterwards, the Click-iT™ Plus EdU Alexa Fluor™ 488 assay was performed according to the manufacturer’s protocol (ThermoFischer). The PCLS were then blocked with 3% BSA solution and incubated with primary anti-ProSPC antibody overnight at 4^o^C, followed by the secondary donkey anti-rabbit Alexa Fluor™ Plus 647 antibody (Invitrogen, ThermoFisher) and fluorescent Cy3-labelled mouse anti- $\:\alpha\:$-Smooth Muscle Actin (αSMA, Merck Millipore) antibody for 1 h at RT. For the imaging, the EVOS M7000 microscope (ThermoFisher) and STELLARIS 5 confocal scanning microscope (Leica Microsystems) were used. Coverslips and customized metal rings (SCIREQ) were used to stabilize the PCLS in 6 well plates. PCLS placed on glass slides were surrounded by imaging spacers (Grace Bio-Labs), with a drop of mounting medium (Agilent Dako) and covered with a microscope cover glass (Marienfield). Analysis was performed using ImagePro 11.1 software (Media Cybernetics) and two customized AI-based protocols (cell count and object within objects). Quantification was performed in at least ten randomly selected images in each PCLS and presented as average value. Proliferating cells were quantified by counting cells that incorporated nucleotide analoque EdU (EdU^+^ cells). ProSPC and αSMA were used markers to count alveolar type 2 and smooth muscle actin containing cells, respectively. The cell number was always related to the total cell count to calculate percentage.

### Statistical analyzes and adjustment of microphotographs

All experiments were performed in at least triplicate unless otherwise stated. The number of independent experiments (n), the method of data generation in the bar and line graphs and the method of statistical analysis of the data are indicated in each figure panel. Brightness and contrast were set uniformly in the photomicrographs in each panel.

## Results

### Development of thermally stable FGF10

**Fig. 1:**
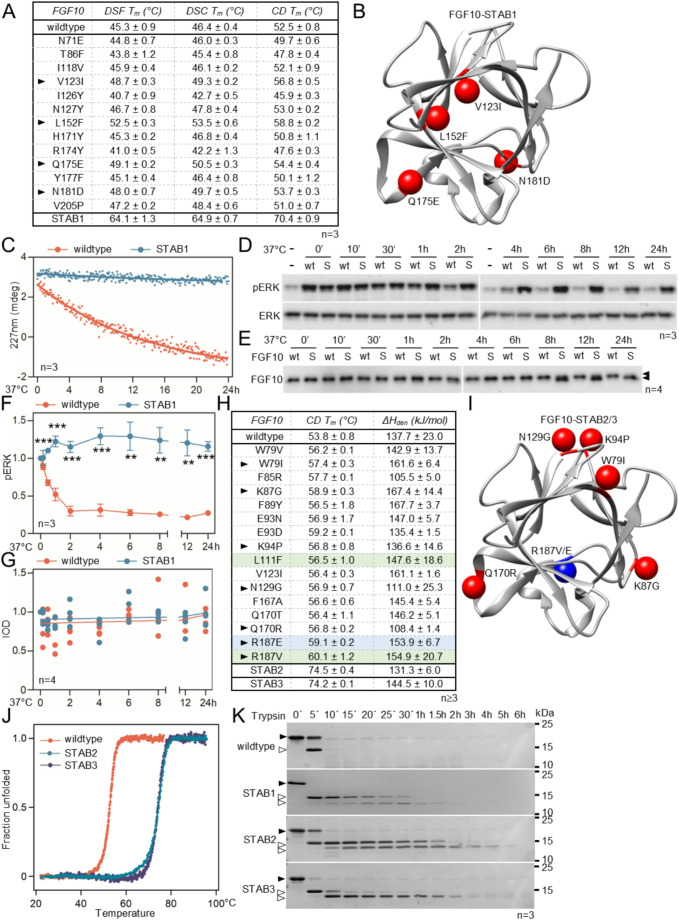
Development of FGF10-STABs. (**A**) Thermal stability of FGF10 variants determined by differential scanning fluorimetry and calorimetry (DSF, DSC) and circular dichroism (CD) spectroscopy. Arrows indicate mutations selected for construction of the combined 4-point mutant designed FGF10-STAB1 (mean ± SD; *T*_*m*_, melting temperature; n, number of independent experiments). (**B**) Model of FGF10-STAB1 with stabilizing substitutions marked in red. (**C**) Thermal stability of the FGF10-STAB1 secondary structure at 37 °C. Changes in FGF10 secondary structure determined by CD spectroscopy by monitoring ellipticity at 227 nm during incubation at 37 °C. Wildtype FGF10 shows unfolding in contrast to FGF10-STAB1. (**D**,** F**) FGF10-STAB1 activity in MCF7 cells. FGF10 variants were pre-incubated at 37 °C for the indicated times before being added to the cells for 30 minutes. Phosphorylated (p) ERK was detected by western blot, with the total amount of ERK serving as a loading control. Densitometric analysis of pERK signal is shown in (F), values relative to cells treated with FGF10 without pre-incubation (0’) (mean ± SEM; Welch *t*-test, ***p < 0.001, **p < 0.01). (**E**,** G**) FGF10 levels after incubation at 37 °C; arrows indicate the double band that appears during incubation of FGF10-STAB1 in culture media. The densitometric analysis of the FGF10 signal is shown in (G). The dots represent individual experimental replicates, the lines are linear regressions showing no difference between wildtype FGF10 and FGF10-STAB1. The data show that the loss of biological activity of wildtype FGF10 (E, G) is caused by thermal unfolding (C) and not by degradation (E, G). (**H**) Development of FGF10-STAB2 and -STAB3. Thermal stability of FGF10 variants determined by CD spectroscopy. Mutations were identified by combination of consensus approach and rational engineering based on 3D structure (white background) and homology strategy (green); mutation impairing binding to heparin is in blue; arrows indicate mutations selected for the construction of the FGF10-STAB2 and -STAB3 (mean ± SEM; H, enthalpy). (**I**) Model of FGF10-STAB2 and -STAB3 with stabilizing substitutions marked in red (blue, R187E/V in STAB2/3). (**J**) Thermal stability of the FGF10-STAB2 and -STAB3 secondary structure at 37 °C. CD spectra show the rapid unfolding of wildtype FGF10 in contrast to the high stability of FGF10-STAB2 and FGF10-STAB3. (**K**) Proteolytic degradation of FGF10 by trypsin. Samples were incubated in the presence of trypsin at 37 °C for up to 6 h and analyzed by SDS-PAGE (black arrowhead indicates full-length FGF10; white arrowheads, products of proteolysis)

In this study, three FGF10 variants with increased thermal stability were developed by two teams using different strategies to select stabilizing point mutations, but working independently. These stable FGF10 variants were designated FGF10-STAB1, FGF10-STAB2 and FGF10-STAB3 (Fig. 1). For FGF10-STAB1, the energy- and evolution-based approaches [26] were used to identify the stabilizing single point mutations in the FGF10 sequence. The in silico design was followed by site-directed mutagenesis and experimental verification of the selected single point mutants. Table S2 summarizes the 14 single point mutations identified by both energy-based and evolution-based approaches. Thirteen mutants were produced in *E. coli* with yields of 10–21 mg per liter of culture. In contrast, FGF10-S143P showed a 90% reduced yield and was excluded from further analyzes. Circular dichroism spectroscopy (CD), differential scanning fluorimetry (DSF) and differential scanning calorimetry (DSC) confirmed the correct folding of all thirteen mutants and an increased melting temperature for six mutants (Fig. 1A).

Four single-point FGF10 mutants with a melting temperature (*ΔT*_*m*_) ≥ 2 °C higher than wildtype FGF10 were combined in silico to form multiple-point mutants (Table S3). The quadruple mutant (V123I, L152F, Q175E, N181D) showed the highest predicted stability and was designated FGF10-STAB1. The mutations are localized in different regions of the FGF10-STAB1 structure, avoiding functionally relevant interfaces such as the FGFR and heparin binding site. Compared to wildtype FGF10, the *T*_*m*_ of FGF10-STAB1 was increased by ~ 19 °C (Fig. 1A, B). This corresponds to the theoretical sum of the individual mutation contribution to overall stability (Δ*T*_*m*_), confirming the additivity of the stabilization effect. We checked the structural integrity of FGF10 by CD and found that the FGF10-STAB1 secondary structure remained stable for at least 24 h at 37 °C, whereas ~ 50% of wildtype FGF10 unfolded within the first 8 h of incubation (Fig. 1C). The improved thermodynamic stability of FGF10-STAB1 resulted in increased resistance to proteolytic degradation; incubation with trypsin caused complete degradation of wildtype FGF10 within 10 min, whereas FGF10-STAB1 was not completely degraded even after 2 h of incubation (Fig. 1K).

Next, we investigated how the increased thermal stability of FGF10-STAB1 correlates with the ability to activate cell signaling. FGF10 was preincubated at 37 °C for up to 24 h before being added to MCF7 breast cancer cells. FGF10-mediated activation of the ERK-MAP kinase pathway was used as a reporter for signal transduction. The biological activity of wildtype FGF10 decreased in about 2 h, in contrast to FGF10-STAB1, which was still fully active after 24 h at 37 °C (Fig. 1D, F). Western blot analysis of FGF10 content in MCF7 culture medium showed that wildtype FGF10 persisted in the culture medium throughout the incubation period (Fig. 1E, G), suggesting that the loss of biological activity of wildtype FGF10 (Fig. 1D, F) was due to the loss of its secondary structure detected by CD (Fig. 1C). FGF10-STAB1 retained its full biological activity after 24 h of incubation at 37 °C.

Template-based thermostability engineering using a consensus approach and rational 3D structure-based engineering was applied for the development of FGF10-STAB2 and FGF10-STAB3. Forty-five FGF10 single-point mutants with increased thermal stability were designed. These mutants were produced in *E. coli* and tested for thermal stability by CD spectroscopy (Fig. 1H, Table S4). Twenty-six mutants showed higher *T*_m_ compared to wildtype FGF10. Using available structures of FGF:FGFR:heparin complexes [35, 36], four additional mutations with predicted lower affinity for heparin were designed (R187E, K191A, R188A, R188E). Subsequently, the stabilizing single point mutations were combined into 21 multiple-point mutants and their thermodynamic stability was analyzed by CD spectroscopy (Table S5). Two stabilizing mutations (L152F, V123I) were identical to those selected for FGF10-STAB1 and were excluded from further analyzes. The combined W79I, K87G, K94P, N129G, Q170R and R187V mutants had a *T*_m_ 20.7 °C higher than that of wildtype FGF10 and were designated FGF10-STAB2 (Fig. 1H, I). The second selected FGF10 variant contained the same substitutions as FGF10-STAB2, with the exception that R187 was replaced by glutamic acid (R187E). This mutant showed a 25% reduction in heparin binding (Table S6) and was labeled as FGF10-STAB3. The improved thermodynamic stability of FGF10-STAB2 and FGF10-STAB3 resulted in increased resistance to proteolytic degradation, as wildtype FGF10 was completely degraded after 10 min of incubation with trypsin, in contrast to FGF10-STAB2 and FGF10-STAB3, which were resistant to trypsin-mediated degradation for more than 1.5 h (Fig. 1K).

Biolayer interferometry (BLI) was applied to verify the interaction of FGF10-STAB2 and FGF10-STAB3 with their cognate receptor FGFR2b [50], using human extracellular domain of FGFR2b, fused with the Fc domain of IgG1. Wildtype FGF10 bound to FGFR2b with K_D_ of 764 nM, which is consistent with the published literature [51] (Fig. S1). The K_D_ for FGF10-STAB2 and FGF10-STAB3 was 996 nM and 913 nM, respectively, indicating that stable FGF10 variants bind to FGFR2b with similar affinity to wildtype FGF10.

### Evaluation of FGF10-STABs in vitro

**Fig. 2:**
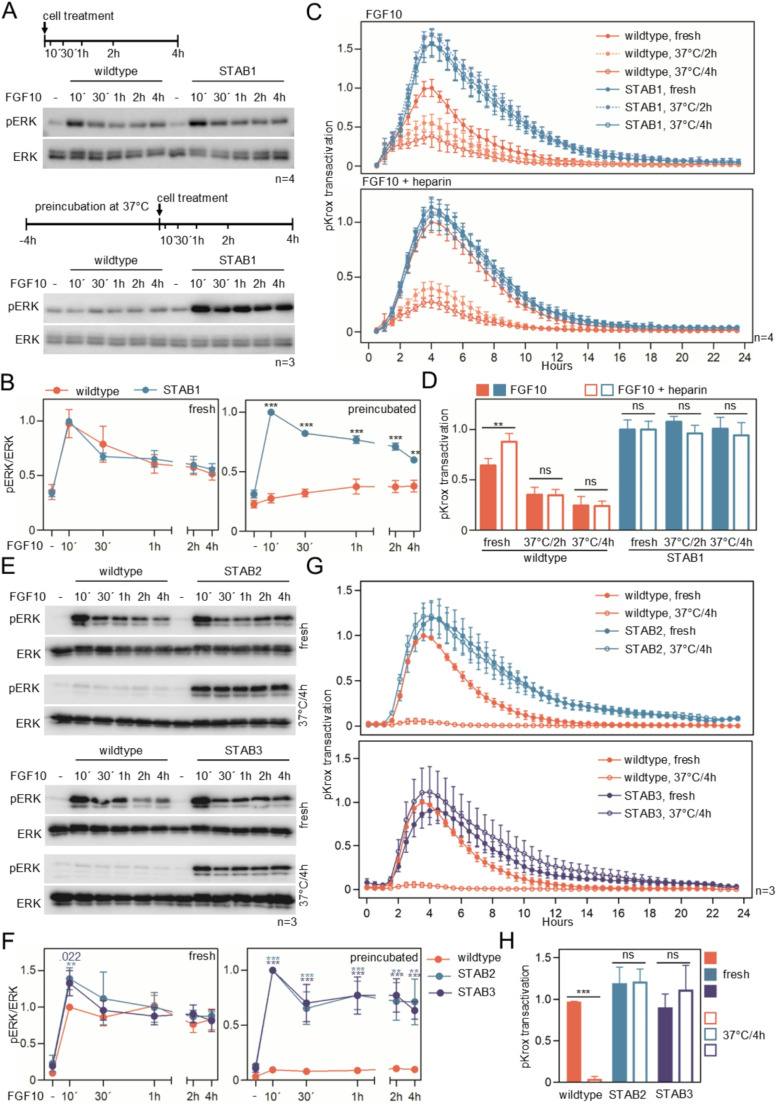
Evaluation of FGF10-STABs in vitro. (**A**, **B**) FGF10-STAB1 activity in 4MBr-5 cells. Cells were treated with fresh (top panel) or with preincubated (37^o^C/4 h) (bottom panel) FGF10 for indicated times. Phosphorylated (p) ERK was detected by western blot; total amounts of ERK serve as loading controls. Densitometric analysis of the pERK signal is shown in (B), data relative to the 10 min FGF10-STAB1 sample (mean ± SEM; Welch *t*-test, ***p < 0.001, **p < 0.01; n, number of independent experiments). (**C**) MCF7^KroxDs^ cells were treated with fresh or preincubated (37^o^C/2–4 h) FGF10 in the absence (top graph) or presence of heparin (bottom graph), and KroxDs transactivation was monitored by automated microscopy for 23 h (mean ± SEM; relative to wildtype FGF10/4 h sample). (**D**) The peak levels of KroxDs transactivation obtained in (C) were plotted. The data show that the addition of heparin improved the activity of fresh but not preincubated wildtype FGF10, indicating that heparin cannot compensate for the poor thermal stability of wildtype FGF10. (**E**, **F**) FGF10-STAB2 and -STAB3 activity in MCF7 cells. Cells were treated with fresh FGF10 (top panel) or with preincubated FGF10 (37^o^C/4 h, bottom panel) and pERK was determined. Densitometric analysis of the pERK signal is shown in (F), data are relative to the 10 min wildtype FGF10 (fresh panel) and FGF10-STAB2/3 (preincubated panel) samples (**G**) MCF7^KroxDs^ cells were treated with fresh or preincubated wildtype FGF10, STAB2 (top graph) and STAB3 (bottom graph), and KroxDs transactivation was monitored for 23 h. (**H**) The peak levels of KroxDs transactivation obtained in (G) were plotted. The data show the loss of wildtype FGF10 activity after preincubation, in contrast to STAB2 and STAB3, which retained full biological activity after 4 h at 37  °C

To gain further insight into FGF10-STAB signaling, 4MBr-5 lung epithelial cells expressing FGFR2b were treated with recombinant FGF10-STAB1 and examined for activation of downstream FGFR signaling. The signaling adaptors FRS2, GAB1 and SHIP2 are the major FGFR substrates in cells and transduce the signal from FGFRs to the downstream signaling pathways, namely ERK-MAP kinase (FRS2, SHIP2) and PI3K/AKT (GAB1) [52, 53]. In 4MBr-5 cells, FGF10 treatment induced phosphorylation of FRS2, GAB1, ERK and AKT (Figs. 2 A; S2A) and no differences in the extent and duration of their phosphorylation were observed between cells treated with wildtype FGF10 and FGF10-STAB1 (Figs. 2B; S2B). Next, we challenged FGF10 by incubating both wildtype and FGF10-STAB1 in complete culture medium at 37 °C for 4 h before addition to the cells. After preincubation, wildtype FGF10 did not elicit any response in 4MBr-5 cells, in contrast to FGF10-STAB1, which activated FRS2, GAB1, ERK and AKT normally (Figs. 2B; S2A, B).

Next, the long-term (up to 23 h) effect of FGF10 on ERK activation was determined. The pKrox24(MapERK)DsRED reporter, developed for monitoring the transcriptional activity of the FGF-ERK pathway [14], was further developed into the less stable variant by replacing the DsRED with destabilized d1EGFP complemented by the 3’-UTR of the mouse Egr1 gene (pKrox24(MapERK)d1EGFP; KroxDs). The MCF7 cells stably expressing KroxDs (MCF7-KroxDs) were treated with FGF10, and the transactivation of KroxDs was monitored for 23 h by automated microscopy. The addition of FGF10 resulted in rapid KroxDs induction in less than 1 h, which persisted up to 10–12 h and peaked at ~ 4 h (Fig. 2C, D). Within the described dynamics, significant differences were observed between wildtype FGF10 and FGF10-STAB1. When fresh wildtype FGF10 was added to the cells, it induced KroxDs to ~ 65% of the level reached with FGF10-STAB1 (Fig. 2D). When FGF10 was challenged by preincubation at 37 °C (2–4 h) prior to addition to the cells, a weaker KroxDs induction of 25 to 33% was observed for wildtype FGF10 compared to FGF10-STAB1 under the same conditions. The stability of the FGF10 secondary structure can be significantly increased by heparin [12]. Figure 2C, D shows that although the addition of heparin increases the activity of fresh wildtype FGF10, it cannot rescue the poor ability of preincubated wildtype FGF10 to trigger KroxDs transactivation.

Similar to FGF10-STAB1, -STAB2 and -STAB3 induced ERK phosphorylation and KroxDs induction in MCF7 cells when preincubated for 4 h at 37 °C, in contrast to wildtype FGF10, which lost its activity during preincubation (Fig. 2E-H). Since FGF10-STAB2 and -STAB3 exhibit reduced heparin binding (Table S6), their ability to induce full ERK activation after preincubation supports the conclusion that stabilization of FGF10 by heparin cannot substitute for the stabilizing mutations. FGF10-STAB2 and -STAB3 were found to be exceptionally stable in the culture media, as they were able to induce ERK activation in MCF7 cells even after 15 days of preincubation at 37 °C (Fig. S3).

Next, we analyzed the FGF10 effect on the modulation of gene transcription. The MCF7 cells were treated with 20 ng/ml of wildtype FGF10 or FGF10-STAB1 and analyzed for changes in gene transcription at 8 h. This time was chosen based on the KroxDS reporter experiments, which show the maximum of KroxDS transactivation at ~ 4 h, with more than 50% of the signal at 8 h (Fig. 2C, G). The KroxDS reporter is based on the promoter of the *Egr1* gene, which represents an early response gene strongly induced by FGF signaling [14]. Thus at 8 h the transcriptional response to the FGF10 was approximately 4 h after the maximum induction of the early response genes. We employed standard RNA-seq procedure and conducted differential gene expression analysis to compare expression profiles between FGF10-STAB1 or wildtype FGF10 treated samples and untreated MCF7 cells. We identified a set of 214 protein-coding genes that were differentially expressed in cells treated with wildtype FGF10 and FGF10-STAB1, when compared to untreated controls. A total of 63 upregulated and 14 downregulated differentially expressed genes exhibited the same trend of deregulation with absolute value of log_2_ fold change > 1.5 in at least one of the comparisons (Table S7). Among the induced transcripts were *ETV4*, *ETV5*, *SPRY4*, *DUSP6* and *EGR3* which are known FGF-response genes [54–58]. Importantly, comparison of transcriptional changes induced by wildtype FGF10 versus FGF10-STAB1 shows virtually identical effect on gene transcription (Fig. S4B, C). Collectively, our data demonstrate that FGF10-STAB1 does not differ from wildtype FGF10 in activation of downstream signaling and induction of the early transcriptional response in cells.

### FGF10-STAB1 induces dimerization and activation of FGFR2b

**Fig. 3:**
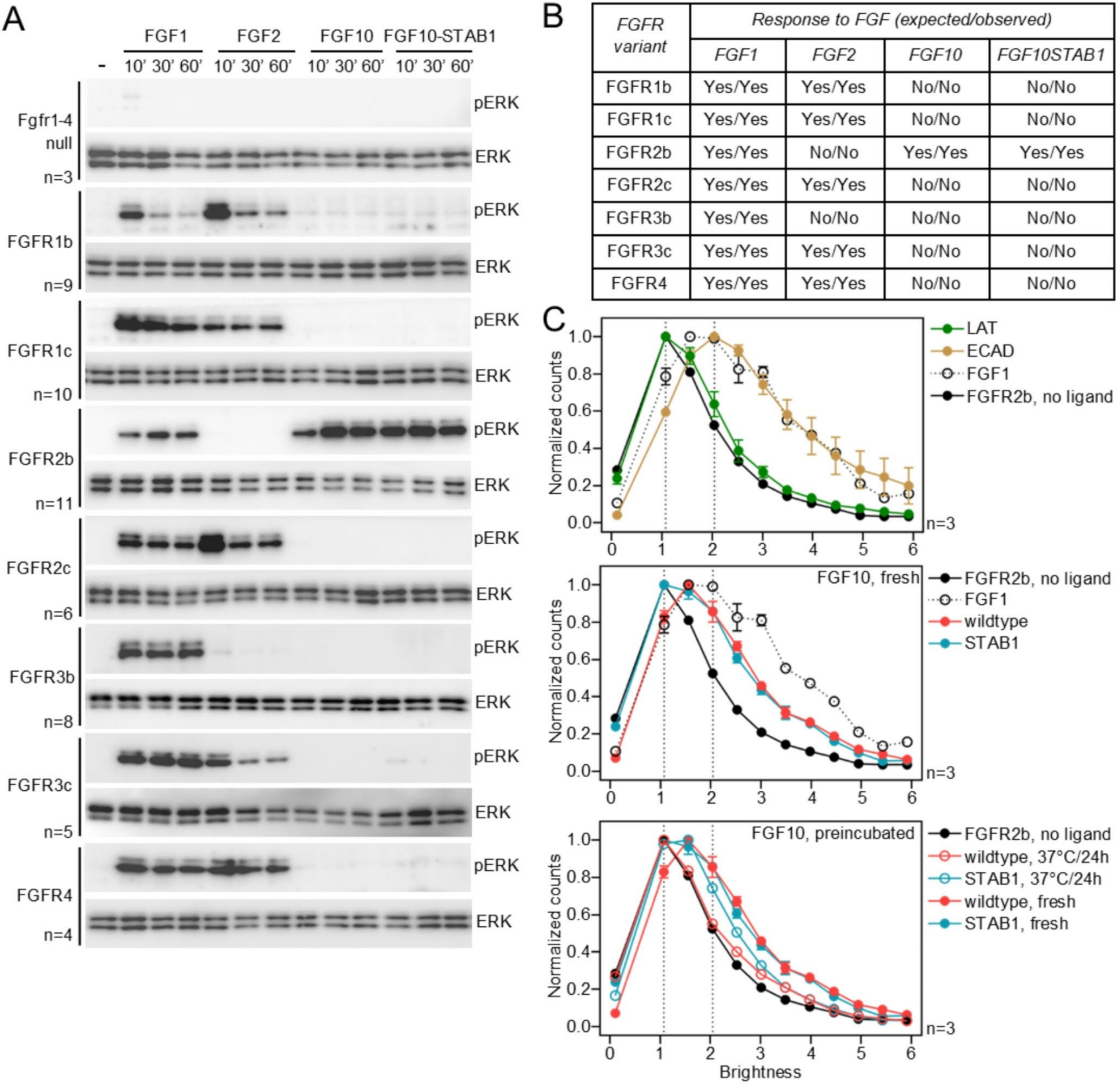
FGF10-STAB1 interaction with FGFRs. (**A**) The RCS cells expressing individual FGFR variants were treated with FGF1, FGF2, wildtype FGF10 and FGF10-STAB1, and analyzed for phosphorylation (p) of ERK; total ERK levels were used as a loading control. (**B**) Summary of the western blot data (B) (obtained), compared to the published data [50, 59]. (**C**) Fluorescence intensity fluctuation (FIF) measurements performed in 293T cells expressing FGFR2b, and treated with FGF10 that had been preincubated at 37 °C for 24 h. Shown are histograms of the measured molecular brightness; FGF1 was used as a positive control of FGFR2b dimerization. Mean ± S.D

The FGFR family consists of four members (FGFR1-4) encoded by four genes that give rise to seven major functional variants. In FGFR1-3, due to alternative splicing, these variants differ in the third immunoglobulin-like domain and are present in two isoforms: “c” and “b”. Since the Ig3 domain contains part of the ligand binding site, the FGFR variants differ in their specificity towards the 22 existing FGF ligands [50, 59]. To investigate the FGFR specificity of FGF10-STAB1, we generated cell lines with defined FGFR expression. Rat chondrosarcoma cells (RCS) are frequently used to study FGF signaling mechanisms [60–63]. In RCS cells, we inactivated the endogenous *Fgfr1-4* genes with CRISPR/Cas9 to generate *Fgfr1-4 null* cells. The *Fgfr1-4 null* cells were stably transfected with vectors containing the individual full-length V5-tagged human FGFRs using PiggyBac transposase for stable integration and an attenuated CMV promoter for low FGFR expression. The resulting seven cell lines expressing single FGFR variants, i.e. FGFR1c, FGFR1b, FGFR2c, FGFR2b, FGFR3c, FGFR3b and FGFR4 [64], were treated with FGF1, FGF2 and FGF10 and FGF-mediated ERK activation was determined (Fig. 3A). Both FGF1 and FGF2 activated FGFR variants according to the expected preferences [50, 59], thus validating the cell models (Fig. 3B). As expected, wildtype FGF10 activated only FGFR2b, similar to FGF10-STAB1. For FGF10-STAB2 and FGF10-STAB3, we verified receptor specificity using the BLI technique. The recombinant extracellular domain of FGFR fused to the Fc fragment was immobilized on a protein A biosensor. The FGFR coupled to the sensor was then used to capture the FGF10. Binding curves for the wildtype FGF10 as well as FGF10-STAB2 and FGF10-STAB3 were very similar (Fig. S5). All FGF10 proteins showed the highest affinity for FGFR2b and a moderate affinity for FGFR1b; no association with other FGFR variants was found. Our data show that stabilizing mutations do not alter the preference of FGF10-STAB1, -STAB2 and -STAB3 for FGFRs.

Next, we compared the ability of wildtype FGF10 and FGF10-STAB1 to stabilize FGFR2b dimers upon binding. We measured the oligomer size of FGFR2b in the absence and presence of the ligands in 293T cells using fluorescence intensity fluctuation (FIF) spectroscopy [39, 40, 65]. To establish the control, we first performed FIF experiments with the monomeric membrane protein LAT [66, 67] and the dimeric membrane protein E-cadherin [68]. FIF spectrometry calculates the molecular brightness of fluorescent protein-tagged receptors in small segments of the plasma membrane and creates a histogram of these molecular brightness values derived from thousands of such segments [65]. The molecular brightness is given by the ratio of the variance of fluorescence intensity within a membrane region to the mean fluorescence intensity in this region and is proportional to the oligomer size. All molecular brightness values measured for LAT and E-cadherin were histogrammed to produce brightness distributions characteristic of monomeric and dimeric controls. We then performed the same experiments with FGFR2b in the absence or presence of FGF1, which is known to induce dimerization and activation of FGFR2b. The FGFR2b variant, in which the intracellular domain was replaced by yellow fluorescent protein, was used. A similar modification has already been successfully used to investigate the self-association of FGFR1 in the plasma membrane [69]. Figure 3C shows that the FIF spectrum for FGFR2b in the absence of the ligand is similar to that of the monomer control LAT. FGFR2b is therefore predominantly monomeric. FGF1 was added at the high saturating concentration of 130 ng/ml to ensure that all FGFR2b molecules were FGF1-bound. After addition of FGF1, the FIF spectra shift to higher brightness values, similar to the dimeric control, as FGF1 induces dimerization of FGFR2b.

Next, we performed experiments with FGFR2b and FGF10 and found distinct differences between the FIF spectra for FGFR2b:FGF1 and FGFR2b:FGF10. The FIF spectra for FGFR2b:FGF10 were intermediate between the FIF spectra of LAT and FGFR2b:FGF1. Under conditions where all FGFR2b molecules are ligand-bound, there is a significant population of FGF10-bound FGFR2b monomers in addition to FGF10-bound FGFR2b dimers. This indicates that FGFR2b:FGF10 is a dimer with lower stability compared to FGFR2b:FGF1. The results were similar for FGF10 and FGF10-STAB1, indicating that stabilization of the ligand does not alter the stability of FGFR2b:FGF10 dimers. Finally, we checked whether preincubation of FGF10 affects the measured FIF spectra. FGF10 and FGF10-STAB1 were incubated at 37 °C for 24 h before addition to the cells. Both FIF distributions decreased in brightness with preincubation time, but the decrease was more pronounced for wildtype FGF10 than for FGF10-STAB1 (Fig. 3C).

Taken together, our data show that FGF10-STAB variants did not differ from wildtype FGF10 in terms of activation of the full diversity of downstream signaling pathways (Figs. 2A, E; S2), signal magnitude and duration (Fig. 2C, G), FGFR specificity (Figs. 3A, B; S5), and induction of early response gene expression in cells (Fig. S4; Table S7). Thus, the increase in thermal stability has only a negligible effect on short-term (up to 24 h) FGF10 signaling.

### FGF10-STAB variants alter cell differentiation in embryonic lung explants

**Fig. 4:**
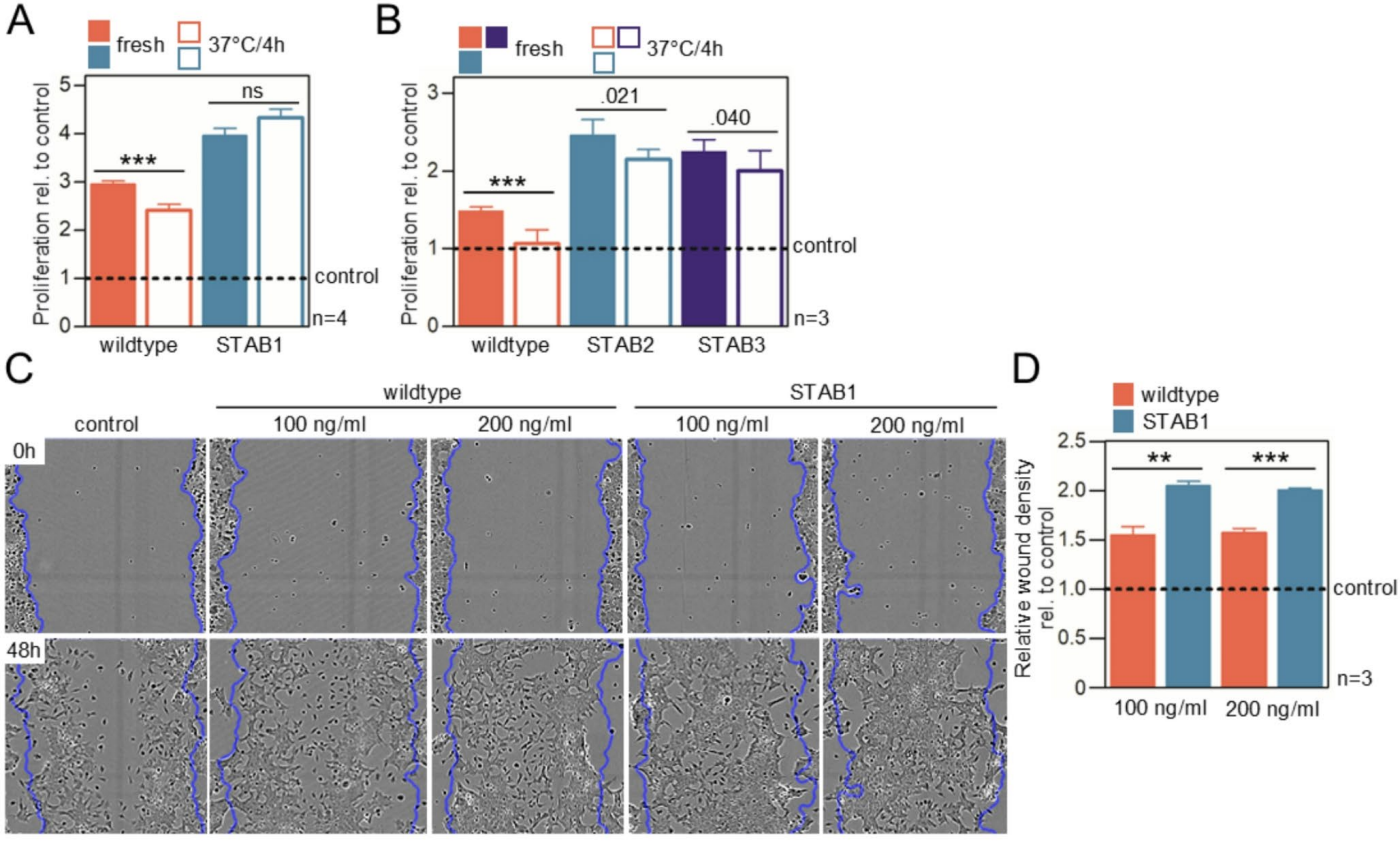
FGF10-STABs accelerate cell proliferation and migration. (**A**, **B**) 4MBr-5 cells were grown for 7 days hours in the presence of FGF10 that was either freshly added to cells, or preincubated at 37 °C for 4 h. Cell proliferation relative to FGF10-naïve cells was plotted (mean ± SEM; Welch *t*-test, ***p < 0.001). (**C**, **D**) FGF10 activity in wound healing assay. MCF7 cells were grown until confluence, scratched and treated with FGF10 for 48 h and the relative wound density was calculated. Normalization of relative wound density was based on both the density of cells in the wound area and the width of the wound itself (mean ± SEM; n, number of independent experiments; unpaired *t*-test, ***p < 0.001, **p < 0.01).

Next, we investigated the effects of FGF10 on cellular processes that require long-term (over 24 h) signaling. MCF7 and 4MBr-5 cells express the FGF10 receptor FGFR2b and respond to FGF10 treatment with activation of ERK and AKT signaling pathways, cytoskeletal remodeling, and increased cell migration (Figs. 1E and 2A and E; S2) [70–73]. The 4MBr-5 cells were used to assess the mitogenicity of FGF10 and the MCF7 scratch assay [74] was performed to investigate the effect of FGF10 on cell migration. In 4MBr-5 cells, a 7-day treatment with FGF10 led to an increase in proliferation, with the effect being more pronounced in cells treated with FGF10-STAB1, -STAB2 and -STAB3 (Fig. 4A, B). Preincubation at 37 °C prior to addition to the cells impaired the ability of wildtype FGF10 to induce proliferation of 4MBr-5, but had no significant effect on FGF10-STABs. In a scratch assay, cell activation and migration led to repopulation of the decellularized area, modeling wound healing. The confluent MCF7 cultures were mechanically scratched and recolonization of the wound area was determined 48 h later (Fig. 4C, D). FGF10 treatment increased the relative wound density compared to the untreated control; this effect was more pronounced in FGF10-STAB1-treated cells at both FGF10 concentrations tested.

**Fig. 5:**
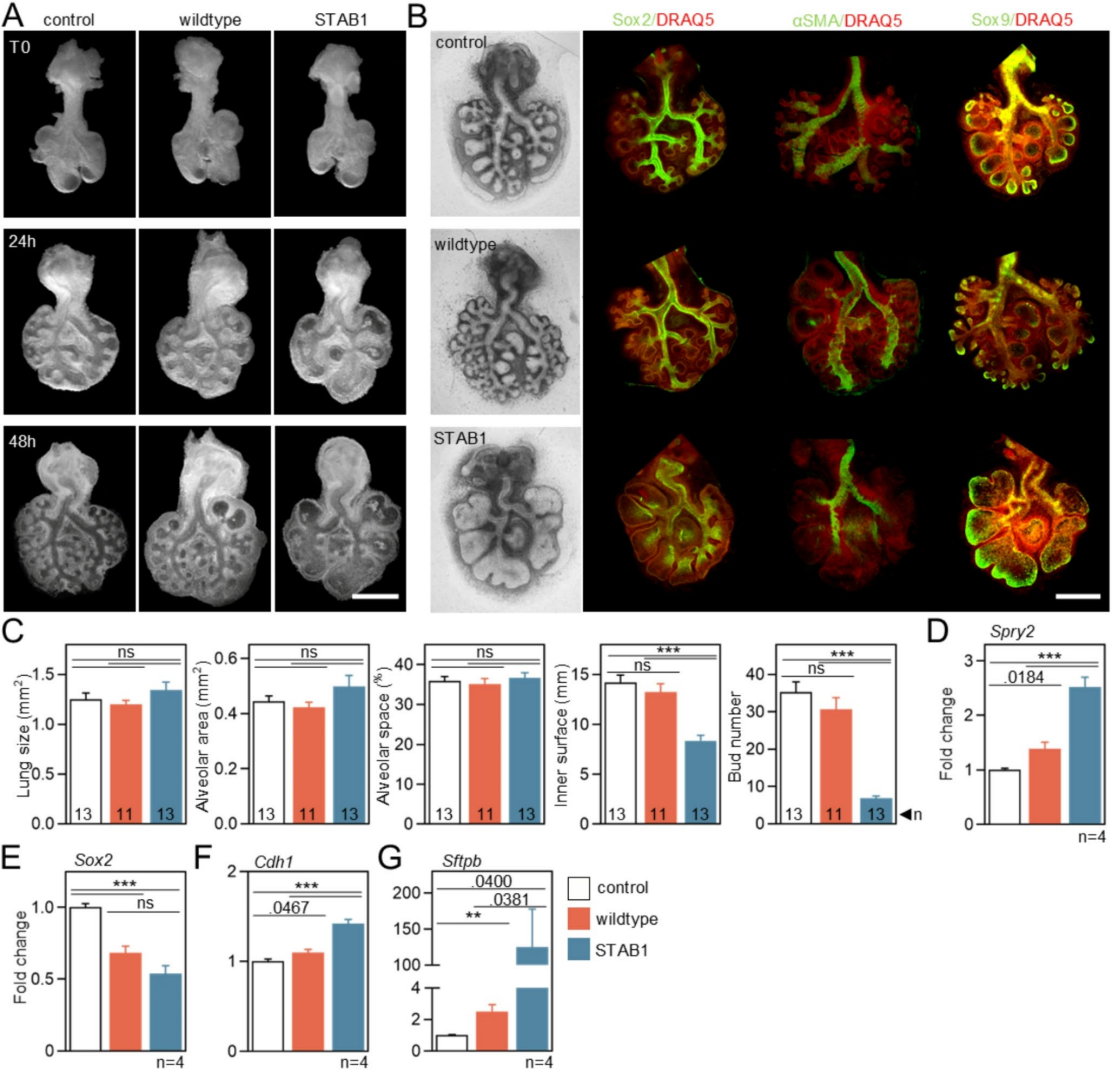
FGF10-STAB1 alters cell differentiation in embryonic lung explant. E11.25 mouse lungs were explanted and incubated for up to 48 h with 500 ng/ml FGF10 added every 12–24 h. (**A**) Overall morphology of lung explants with branching suppressed by FGF10-STAB1 (scale bar, 0.5 mm). (**B**) Whole mount immunohistochemistry of explants grown for 60 h. Antibodies for SOX9 (distal epithelial progenitors), SOX2 (proximal epithelial progenitors) and αSMA (parabronchial smooth muscle progenitors) were used together with DRAQ5 as nuclear counterstain (scale bar, 0.5 mm). (**C**) Lung size, alveolar area, alveolar space, inner surface and buds number of explants grown for 60 h with FGF10-STAB1 (mean ± SEM, One-Way ANOVA, ***p < 0.001, **p < 0.01). (**D**) Quantitative RT-PCR (qPCR) analysis of FGF10 transcriptional target *Spry2* in explants grown for 60 h; data normalized to *Actb* expression. (**D**-**F**) qPCR analysis of expression of *Sox2*,* Cdh1* (epithelial lineage) and *Sftpb* (distal epithelial progenitors) (mean ± SEM of four experiments with two explants each (Welch *t*-test, ***p < 0.001, **p < 0.01)

During lung formation, the FGF10:FGFR2b signaling axis regulates epithelial proliferation and cell lineage commitment during the branching stage, which is essential for proximal-distal patterning and shaping of branched structures [75, 76]. To investigate the effect of FGF10-STAB on lung development, mouse embryonic lung explants were isolated at the early pseudoglandular stage (E11.25) and cultured in the presence of FGF10 for 48–72 h. Treatment with 500 ng/ml FGF10-STAB1 resulted in notable morphological changes in the developing lung, manifested by significant dilation of the distal epithelium, lack of branching of both proximal and distal epithelial buds, and swelling of the buds into cyst-like structures (Figs. 5A; S6). In contrast, 500 ng/ml wildtype FGF10 caused only a weak morphological effect on proximal buds and no effect on distal buds (Figs. 5A-C, S6). Evaluation of the FGF10-STAB1 concentration range revealed that a significant decrease in buds number at 200 and 500 ng/ml, while no effect was found at 100 ng/ml (Fig. S7). A corresponding, but weaker effect was observed for FGF10-STAB2 (Fig. S8). The same treatments with FGF10-STAB3 showed a significant drop of buds number starting at 100 ng/ml (Fig. S9). Moreover, FGF10-STAB3 significantly reduced the inner alveolar surface at a concentration of 100 ng/ml, while the effects of STAB1 and STAB2 were milder (starting from a concentration of 200 ng/ml) (Figs. S7-9). These morphological changes resulted in a significant dilation of epithelial buds.

To gain more insight into the cellular processes underlying the morphological changes induced by FGF10-STABs, we examined the cell lineages contributing to lung explant development. Expression of the FGF10 transcriptional target *Spry2* [77] was induced in lung explants treated with wildtype FGF10 and FGF10-STAB1 (Fig. 5D), but was more pronounced in the latter. FGF10 treatment modulated both epithelial and mesenchymal differentiation, as shown by whole-mount immunohistochemistry for markers of proximal epithelium (SOX2), distal epithelium (SOX9) and airway smooth muscle cells (αSMA). The number of SOX2^+^ and αSMA^+^ cells was reduced by the addition of FGF10 (Fig. 5B), indicating a general suppression of differentiation into proximal cell types. FGF10-STAB1 showed a stronger inhibitory effect compared to wildtype FGF10. A significant reduction of SOX2^+^ cells in FGF10-STAB1-treated lungs was confirmed by analyzing *Sox2* gene expression (Fig. 5E). The main morphological change induced by FGF10-STAB1, -STAB2 and -STAB3 was a significant expansion of the distal epithelium of the lung buds, causing bud swelling into cyst-like structures (Figs. 5A-C, S6-9). This was likely caused by the expansion of distal epithelial progenitors, as evidenced by the increased number of SOX9^+^ cells and induction of E-cadherin (*Cdh1*) expression in FGF10-STAB1-treated lungs (Fig. 5C, F). Further epithelial differentiation was also increased by FGF10-STAB1, as shown by the induction of alveolar type II cell marker surfactant protein b (*Sftpb*) expression (Fig. 5G).

### FGF10-STAB1 increases cell differentiation in human iPSC-derived lung organoids (LOs)

**Fig. 6:**
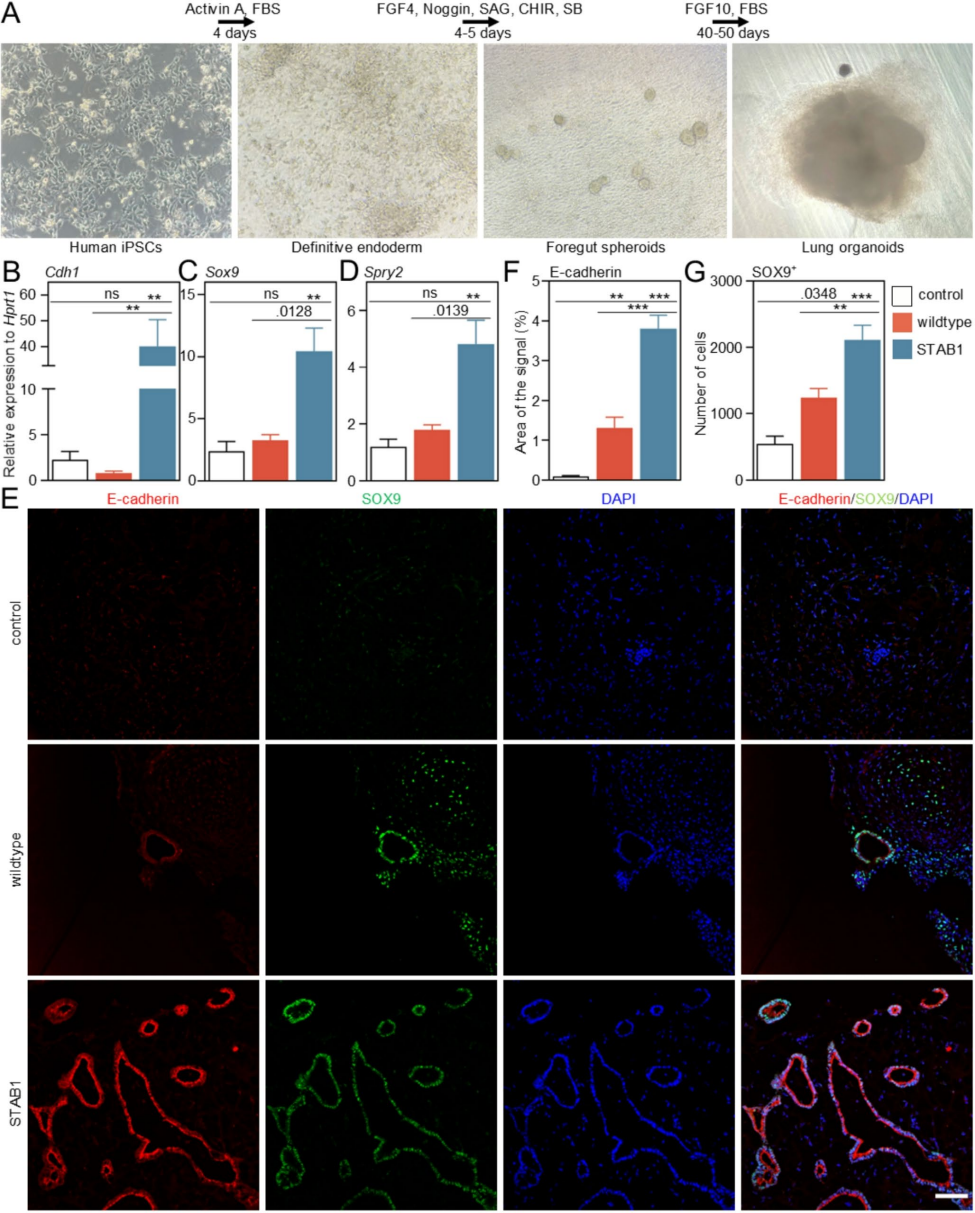
FGF10-STAB1 affects cell differentiation in human iPSC-derived lung organoids (LOs)(**A**) Formation of human iPSC-derived lung organoids. (**B**-**D**) RNA expression of *Cdh1*, *Sox9*, and *Spry2* in LOs differentiated with wildtype FGF10 or FGF10-STAB1 determined with quantitative PCR (qPCR), gene expression was normalized to *Hprt1*. LOs differentiated without FGF10 were used as control (*n* = 3). (**E**) Immunofluorescence staining of E-cadherin and SOX9 counterstaining with DAPI, scale bar 100 μm, three independent biological replicates were used. (**F**) Quantification of E-cadherin positive area in the immunofluorescence staining of LOs, three independent biological replicates were used with at least two technical replicates (different parts of the same organoid; *n* = 8–10). (**G**) Quantification of SOX9 cells in the immunofluorescence staining of LOs, three independent biological replicates were used with at least two technical replicates (different parts of the same organoid; *n* = 8–10) (bar plots, mean ± SEM; one-way ANOVA with Tukey’s multiple comparison test was used to determine statistical significance, ***p < 0.001, **p < 0.01)

We employed human induced pluripotent stem cell (iPSC)-derived lung organoids (LOs) (Fig. 6A) to evaluate the effect of FGF10-STAB on lung differentiation. Previously, we have shown that iPSC-derived LOs form complex organized structures consisting of epithelial and mesenchymal cells and thus allow study structural and cell population changes upon different treatment [44]. First, using qPCR, we showed a significant increase of *Cdh1*, *Sox9* and *Spry2* (Fig. 6B-D) expression in LOs differentiated with FGF10-STAB1 compared to wildtype FGF10 and control without FGF10. Immunofluorescence was used to describe the effect of FGF10-STAB1 on epithelial (E-cadherin^+^) cells and distal epithelial progenitors (SOX9^+^) (Fig. 6E). We observed an increased number of epithelial cells as demonstrated by significantly increased area of E-cadherin staining (Fig. 6F) in LOs differentiated with FGF10-STAB1 compared to wildtype FGF10 and control without FGF10. Furthermore, the quantification revealed a significant increase of SOX9^+^ cells within the lung tissue in LOs differentiated with FGF10-STAB1 compared to wildtype FGF10 and control without FGF10 (Fig. 6G).

### FGF10-STAB2 reduces fibrosis and regenerates epithelia in ex vivo mouse model of lung injury

**Fig. 7:**
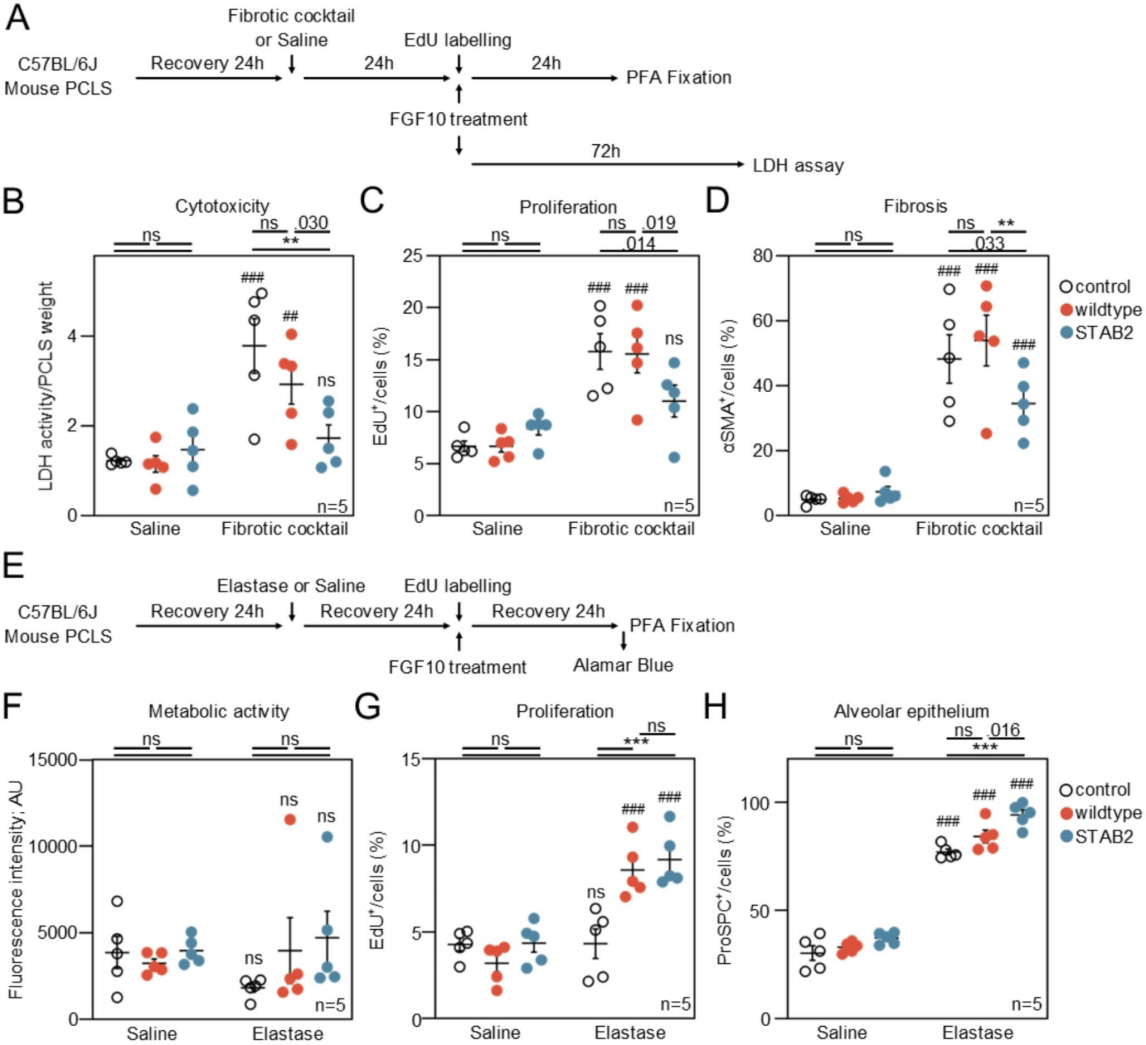
FGF10-STAB2 reduces fibrosis and regenerates epithelia in an ex vivo mouse model of lung injury. (**A-D**) Ex vivo fibrotic cocktail-induced early fibrosis mouse precision-cut lung slices (PCLS) model. (**A**) Experimental design and timeline. (**B**) Cytotoxicity in saline or fibrotic cocktail PCLS upon 72 h treatment with vehicle, 250 ng/ml of wildtype FGF10 or FGF10-STAB2. (**C**) Analysis of proliferation (EdU^+^) and (**D**) alpha-smooth-muscle-actin positive (αSMA^+^) cells. (**E-H**) Ex vivo model of elastase-induced injury in mouse PCLS. (**E**) Experimental design and timeline. (**F**) Metabolic activity in saline or elastase PCLS after 24 h treatment with vehicle, wildtype FGF10 or FGF10-STAB2 (250 ng/ml). (**G**) Analysis of proliferating (EdU^+^) and (**H**) alveolar epithelial type 2 (ProSPC^+^) cells. Each point represents a biological replicate (n); two-way ANOVA, ***p < 0.001, **p < 0.01; ###<0.001, ##<0.01 (comparison with corresponding control animals)

The potential regeneration effect of stable FGF10 was verified using two ex vivo mouse models, an early fibrosis-like changes model [47] and an elastase model mimicking the COPD-relevant phenotype [45, 48]. To induce fibrosis or lung injury, mouse precision-cut lung slices (PCLS) were incubated 24 h with a fibrosis cocktail or elastase, respectively, and then treated with 250 ng/ml of wildtype FGF10 or FGF10-STAB2 (Fig. 7A, E) for 24–72 h for accessing metabolic activity/imaging or cytotoxicity, respectively.

In early-fibrosis PCLS, the release of LDH (lactate dehydrogenase) from cells was decreased by FGF10-STAB2 compared to wildtype FGF10 and to saline, suggesting reduced cytotoxicity in PCLS induced by stable FGF10 variant (Fig. 7B). In line with the decreased cytotoxicity, proliferation rate of the cells and number of αSMA^+^ cells, which are hallmarks for pulmonary fibrosis were limited by FGF10-STAB2, whereas wildtype FGF10 showed no effect (Figs. 7C, D; S11A). In elastase-induced emphysema model, both wildtype and STAB2 variants of FGF10 increased cell proliferation with no impact on metabolic activity (Figs. 7F, G; S11B). When compared to wildtype FGF10, the treatment with FGF10-STAB2 led to increased amounts of ProSPC^+^ cells, a marker for progenitor alveolar type II (AT2) cells (Fig. 7H) [49]. Altogether, the data suggest that FGF10 increases regeneration in injured (fibrotic cocktail or elastase) but not in control PCLS and that FGF10-STAB2 has enhanced regenerative properties when compared to wildtype FGF10.

### FGF10-STAB1 affects limb development

**Fig. 8:**
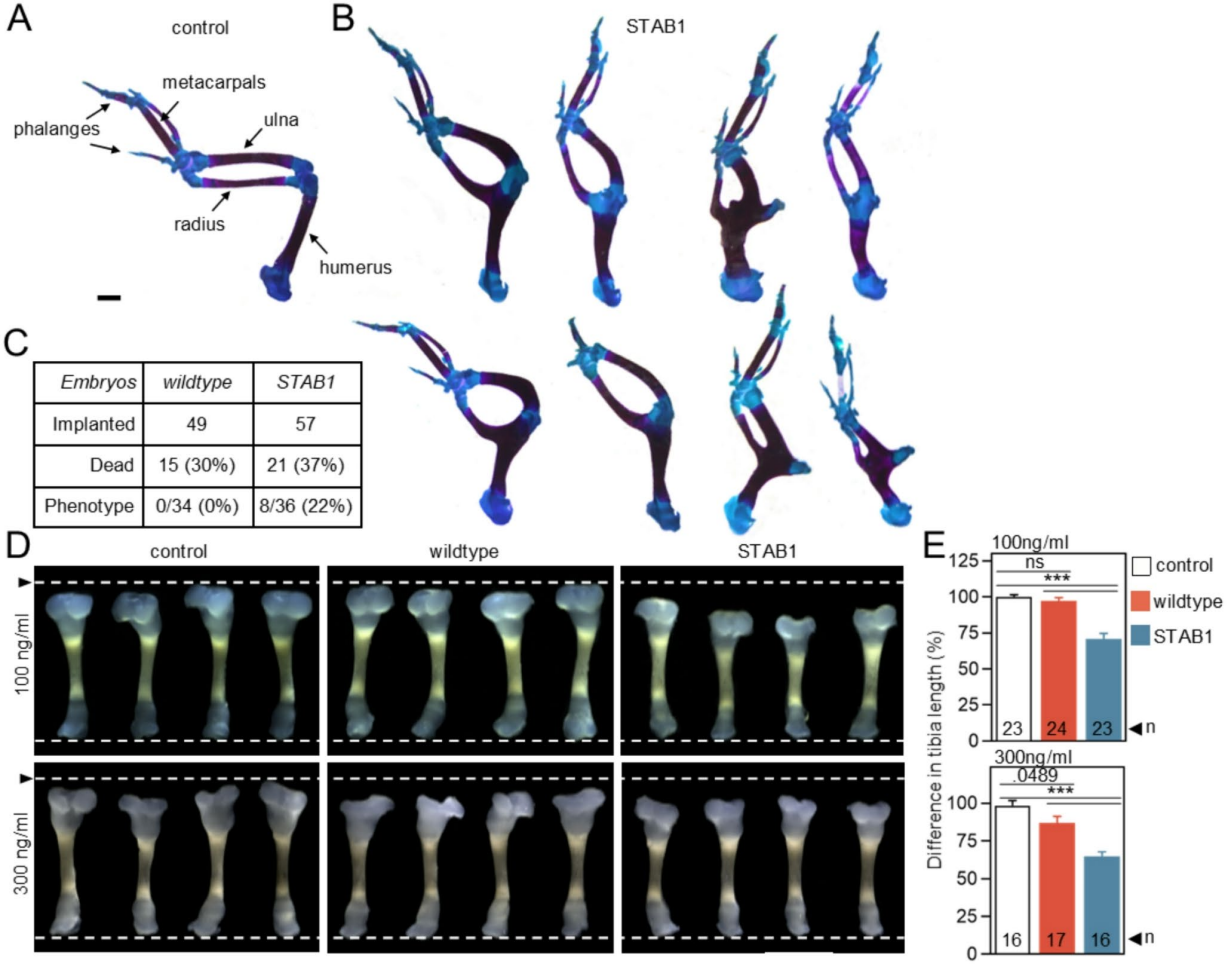
FGF10-STAB1 affects endochondral ossification. (**A**-**C**) Affigel blue beads soaked with 1 mg/ml FGF10 were implanted into chick embryonic forelimb buds at HH19-21; the limb phenotype was analyzed 10–12 days later. The beads were implanted only in the right buds, while left buds served as controls. (**A**) Normal limb skeleton at the end of the experiment, stained with Alcian Blue and Alizarin Red (scale bar, 1 mm). (**B**) Overall appearance of eight limb skeletons treated with FGF10-STAB1; no malformations were observed in animals implanted with wildtype FGF10 (not shown). (**C**) Summary of implantation experiments showing the number of FGF10-treated embryos, overall survival rate and the incidence of limb deformities (phenotype). (**D**, **E**) E18 mouse tibia were cultured with 100 ng/ml and 300 ng/ml FGF10 for 8 days. (**D**) Overall appearance of the tibia at the end of the experiment. Images are representative of three independent experiments (arrow, length of average untreated tibia). (**E**) Differences in tibial length before and after 8 days of cultivation (mean ± SEM; n, number of tibia; Welch *t*-test, ***p < 0.001, **p < 0.01)

FGF10 is a key regulator of early limb development that is important for the initiation and maintenance of limb bud outgrowth [78]. First, the effect of FGF10-STAB1 on early limb development was investigated. Agarose beads soaked with wildtype FGF10 and FGF10-STAB1 (1 mg/ml) were implanted into chicken embryonic limbs (right wing) at the bud stage (HH19-21) [12]; the left wing was used as non-treated control. Chicken embryos were incubated for an additional 10–12 days (HH38) to allow for complete skeletal development, and skeletal elements were stained with Alcian Blue and Alizarin Red to assess changes in the humerus, ulna, radius, metacarpals, and phalanges. Macroscopic analysis revealed shortened right wings in 8/36 (22%) animals treated with FGF10-STAB1 exhibiting significant limb deformities, including shortening of the long bones, particularly in the zeugopodial and stylopodial zones, and fusions in the joint areas (Fig. 8A-C). No abnormalities were detected in 34 animals treated with wildtype FGF10.

Next, we examined the effects of FGF10 on the growth of skeletal elements undergoing endochondral ossification and containing both the growth plate cartilage and bone components. Tibial explants isolated from E18 mouse embryos were cultured for 8 days in media supplemented with wildtype FGF10 and FGF10-STAB1, and tibial length was determined before and after cultivation (Fig. 8D, E). FGF10-STAB1 caused a significant reduction in tibial length at both 100 ng/ml (percentage of control ± SEM, 70.9 ± 3.8%) and 300 ng/ml (64.8 ± 3.1%) (Fig. 8D, E). Tibias treated with wildtype FGF10 showed no growth inhibition at 100 ng/ml (97.2 ± 2.3%; not significant) and a weakly significant inhibition at 300 ng/ml (86.8 ± 4.6%; *p*=0.0489) compared to untreated control tibias.

## Discussion

Experimental in vitro studies show that the thermal stability and resistance to proteolytic degradation vary widely among mammalian FGFs [11, 12]. While the endocrine FGFs, FGF19, FGF21 and FGF23, remain stable for at least 8 h when exposed to cell-conditioned media at 37 °C, other FGFs exist as unstable proteins that are rapidly degraded in less than one hour under the same conditions. The limited stability appears to restrict the signaling of at least 11 FGFs (FGF1/3/4/6/8–10/16/17/20). We have previously used site-directed mutagenesis to improve the resistance of FGF1 and FGF2 secondary structure to thermal unfolding. For example, a three-point mutant of FGF1 showed increased thermal stability by 21 °C and significantly improved biological activity in vitro without affecting essential functions such as proteoglycan or FGFR binding [9, 79]. Similarly, a nine-point mutant of FGF2 was developed that has a 19 °C higher melting temperature and greatly increased biological activity compared to wildtype FGF2 [29]. FGF4 and FGF18 have also been improved by mutations that increase thermal stability [80, 81]. The fact that thermal instability affects at least 11 of the 22 known mammalian FGFs suggests that stability is a key issue in FGF biology. Nevertheless, the question of the physiological role of FGF stability has never been addressed; we know nothing about the role of limited FGF stability in vivo.

Among the mammalian morphogen families, the TGFβ/BMP (transforming growth factor beta/bone morphogenic protein) and WNT (wingless and int) systems resemble the FGF family in terms of the high variability of existing extracellular ligand-receptor interactions. In FGF signaling, 22 FGFs activate seven FGFR variants, while in the WNT family, 20 WNT ligands activate 10 cognate receptors (excluding co-receptors) [82]; in mammals, there are more than 30 TGFβ/BMP ligands belonging to the TGFβ, BMP, GDF (growth and differentiation factor), nodal and activin families [83]. These ligands signal by interacting with a heterotetrameric receptor complex consisting of type I/type II receptors. Seven type I and five type II receptors are known [84].

In both the WNT and TGFβ/BMP systems, ligand-receptor interactions are regulated by a significant number of secreted extracellular antagonists that sequester ligands and thus prevent binding to their cognate receptors or neutralizing the receptors. More than 10 such secreted antagonists have been identified in the WNT signaling pathway, including four dickkopf proteins (DKK1-4), five sFRP proteins (sFRP1-5), WIF1, and others [85]. There are more than 10 secreted antagonists in the TGFβ/BMP signaling pathway, such as noggin, chordin, follistatin, gremlin, cerberus and others [86]. Secreted antagonists exert a key role in regulating morphogen function by restricting signaling in scenarios where morphogen action is no longer required, is excessive, or is at risk of causing a defect through ectopic signaling.

In contrast, extracellular FGF-FGFR interactions do not appear to be modulated by secreted antagonists, although R-spondin 2 was recently shown to bind FGFR4 and neutralize FGFR4 signaling during body axis formation in Xenopus [87]. To our knowledge, no other secreted extracellular antagonists of FGF signaling in development have been identified. We hypothesize that this is due to the low stability of FGF proteins. At 37 °C, the FGF tertiary structures exist in a partially unfolded state, which prevents binding to FGFR and activation of FGFR signaling. The interaction with proteoglycans has a well-documented stabilizing effect on FGF structure [12, 88, 89]. Since proteoglycans are mostly anchored in the cell membrane, the unstable FGFs can only signal locally after they have been stabilized by interacting with proteoglycans on the cell surface. This reduces the risk of ectopic signaling to distant cells, as the FGFs unfold and are degraded before they reach them, presumably making the existence of a secreted extracellular FGF antagonist unnecessary.

We hypothesized that instability is a strong obstacle to prevent ectopic FGF signaling in complex developmental scenarios. FGF10 was selected because it was shown to be one of the least stable FGFs in our previous study [12]. In this study, we produced three stable FGF10 variants developed independently by two teams that exhibited thermal stability improved by more than 19 °C, while containing non-overlapping stabilizing mutations to avoid potential artefacts caused by changes in the primary protein sequence (Fig. 1). We show that FGF10-STAB variants did not differ from wildtype FGF10 in their specificity for seven existing FGFR variants, ability to bind the cognate receptor FGFR2b, activation of downstream FGFR2b signaling or induction of gene transcription (Figs. 2 and 3, S2, S4, S5; Table S7). However, in long-term experiments such as cell proliferation and scratch assay the FGF10-STAB variants demonstrated greater biological activity than wildtype FGF10 (Fig. 4). Experimental models of lung and limb development were selected to investigate the putative ectopic signaling of FGF10-STAB, as they represent the two major areas of FGF10 morphogenic signaling in vivo.

Deletion of FGF10 leads to failure of lung development due to impaired branching morphogenesis. In *Fgf10*^*−/−*^ mouse embryos, the rudiments of the trachea and lung buds are retained, suggesting that FGF10 stimulates lung bud growth immediately after initiation [7, 90]. Later, at the early pseudoglandular stage of lung development (E10.5-E12.5), FGF10 regulates proximal-distal differentiation along the alveolar stalk [75]. Previous experimental studies have shown that FGF10 expressed in lung mesenchyme regulates proliferation and differentiation of epithelial cells expressing FGFR2b, in contrast to FGF9 expressed by epithelial and mesothelial cells [91], and that it signals via FGFR3b to promote distal epithelial fate specification and inhibit epithelial differentiation [92]. Our data are consistent with this model. We show that experimental upregulation of FGF10 signaling in lung explants treated with FGF10-STAB results in suppressed branching and significant epithelialization of the lung by increasing distal epithelial cell proliferation and differentiation (Figs. 5, S6-9). Thus, limited FGF10 stability appears to contribute to the proper dose of FGF10:FGFR2b signaling required to maintain the balance between epithelial branching, proximal-distal specification and differentiation required for proper lung development.

During early embryonic limb development, a bidirectional FGF signaling pathway mediates communication between ectoderm and mesenchyme in the limb bud [93], whereby FGF4 and FGF8 are expressed in the apical ectodermal ridge (AER) at the distal tip of the limb bud and activate FGFR1c in the underlying mesenchyme. The mesenchyme expresses FGF10, which signals to FGFR2b, which is expressed in the ectoderm. FGF8 and to some extent FGF4 regulate cell number in the developing limb bud and are required for the survival of cells located far from the AER, as has been shown by the failure of limb development in *Fgf4/Fgf8*-deficient mice [94]. The *Fgf10 null* animals also fail to develop limbs because the epithelial-to-mesenchymal transition of the coelomic epithelium, which is required for limb bud growth immediately after limb induction, is impaired [7, 95]. Experimental exposure of chick limb buds by of FGF10-STAB1, but not wildtype FGF10, resulted in significant deformities of the resulting limb skeleton, including shortening of the long bones and joint fusions (Fig. 8B, C). It should be noted that these deformities affected only 22% of the embryos treated at stage HH19-21. We assume that this is due to the increasing size of the limb bud at HH stages 20 and higher preventing FGF10 from reaching the recipient cells. This is suggested by analysis of FGF10 expression in HH20+, which is restricted to the mesenchymal tissue immediately beneath the ectoderm, therefore the proximal and central zones of the limb bud are devoid of FGF10 [96, 97]. If the FGF10 soaked bead is implanted into this region, it is unlikely that the recombinant FGF10 will reach the target cell in the ectoderm. Nevertheless, our data provide clear evidence for ectopic signaling induced by FGF10-STAB1.

Following the collection of evidence for ectopic signaling of FGF10-STAB1 in the two areas of physiological FGF10 function, we examined the effect of FGF10 on the growth of E18 mouse tibial explants already containing differentiated bone and growth plate cartilage. Long bone growth does not appear to be the main area of FGF10 function in development. First, growth plate cartilage does not express *Fgf10* [98]. Second, the chondrocytes are of mesenchymal origin and therefore express the ‘c’ variant of FGFR3, which is not a cognate FGF10 receptor [50, 99]. FGF10 inhibited cell proliferation in human growth plate chondrocyte cultures [100] and induced shortening of skeletal elements in a transgenic mouse model [101], although the relevance of these findings is unclear. While the first effect may be considered an artifact of tissue culture, the second effect may be due to a developmental rather than a growth plate cartilage defect. However, our tibial explant experiment revealed that FGF10-STAB1, but not wildtype FGF10, can signal even outside its physiological domain of function and affect cartilage tissue that should not normally respond to FGF10 signal (Fig. 8D, E).

Overall, our results suggest that the thermal instability of FGF10 represents a strong barrier to uncontrolled signaling during development. Therefore, FGF instability should be considered when interpreting FGF functions in vivo, especially for FGF8, which is a key factor in midbrain and hindbrain patterning and limb morphogenesis [94, 102] and, similar to FGF10, is a highly unstable protein [12]. Finally, we demonstrate enhanced lung differentiation of human iPSC-derived lung organoids, and increased regeneration in lung injury models mediated by FGF10-STAB variants developed here. This opens up the attractive possibility of using FGF10-STAB in cell therapy aimed at restoring lung function.

## Electronic supplementary material

Below is the link to the electronic supplementary material.

Supplementary Material 1

## Data Availability

All data generated or analyzed in the study are available from the corresponding authors on reasonable request.

## References

[1] Itoh N, Ornitz DM (2008) Functional evolutionary history of the mouse *Fgf* gene family. Dev Dyn 237:18–27. 10.1002/dvdy.21388 18058912 10.1002/dvdy.21388

[2] Colvin JS, Green RP, Schmahl J et al (2001) Male-to-Female sex reversal in mice lacking fibroblast growth factor 9. Cell 104:875–889. 10.1016/S0092-8674(01)00284-7 11290325 10.1016/s0092-8674(01)00284-7

[3] Ohbayashi N, Shibayama M, Kurotaki Y et al (2002) FGF18 is required for normal cell proliferation and differentiation during osteogenesis and chondrogenesis. Genes Dev 16:870–879. 10.1101/gad.965702 11937494 10.1101/gad.965702 PMC186331

[4] Usui H, Shibayama M, Ohbayashi N et al (2004) Fgf18 is required for embryonic lung alveolar development. Biochem Biophys Res Commun 322:887–892. 10.1016/j.bbrc.2004.07.198 15336546 10.1016/j.bbrc.2004.07.198

[5] Lu SY, Sontag DP, Detillieux KA, Cattini PA (2008) FGF-16 is released from neonatal cardiac myocytes and alters growth-related signaling: a possible role in postnatal development. Am J Physiol-Cell Ph 294:C1242–C1249. 10.1152/ajpcell.00529.2007 10.1152/ajpcell.00529.2007 PMC5224940 18337564

[6] Cholfin JA, Rubenstein JLR (2007) Patterning of frontal cortex subdivisions by Fgf17. P Natl Sci 104:7652–7657. 10.1073/pnas.0702225104 10.1073/pnas.0702225104 PMC1863435 17442747

[7] Sekine K, Ohuchi H, Fujiwara M et al (1999) Fgf10 is essential for limb and lung formation. Nat Genet 21:138–141. 10.1038/5096 9916808 10.1038/5096

[8] Zakrzewska M, Krowarsch D, Wiedlocha A, Otlewski J (2004) Design of fully active FGF-1 variants with increased stability. Protein Eng Des Sel 17:603–611. 10.1093/protein/gzh076 15469994 10.1093/protein/gzh076

[9] Zakrzewska M, Krowarsch D, Wiedlocha A et al (2005) Highly stable mutants of human fibroblast growth factor-1 exhibit prolonged biological action. J Mol Biol 352:860–875. 10.1016/j.jmb.2005.07.066 16126225 10.1016/j.jmb.2005.07.066

[10] Zakrzewska M, Wiedlocha A, Szlachcic A et al (2009) Increased protein stability of FGF1 can compensate for its reduced affinity for heparin. J Biol Chem 284:25388–25403. 10.1074/jbc.M109.001289 19574212 10.1074/jbc.M109.001289 PMC2757240

[11] Chen G, Gulbranson DR, Yu P et al (2012) Thermal stability of fibroblast growth factor protein is a determinant factor in regulating Self-Renewal, differentiation, and reprogramming in human pluripotent stem cells. Stem Cells 30:623–630. 10.1002/stem.1021 22213113 10.1002/stem.1021 PMC3538808

[12] Buchtova M, Chaloupkova R, Zakrzewska M et al (2015) Instability restricts signaling of multiple fibroblast growth factors. Cell Mol Life Sci 72:2445–2459. 10.1007/s00018-015-1856-8 25854632 10.1007/s00018-015-1856-8 PMC11113989

[13] Miller DL, Ortega S, Bashayan O et al (2000) Compensation by fibroblast growth factor 1 (FGF1) does not account for the mild phenotypic defects observed in FGF2 null mice. Mol Cell Biol 20:2260–2268. 10.1128/MCB.20.6.2260-2268.2000 10688672 10.1128/mcb.20.6.2260-2268.2000 PMC110842

[14] Gudernova I, Foldynova-Trantirkova S, El Ghannamova B et al (2017) One reporter for in-cell activity profiling of majority of protein kinase oncogenes. Elife 6. 10.7554/eLife.21536 10.7554/eLife.21536 PMC5310841 28199182

[15] Hansson EM, Teixeira AI, Gustafsson MV et al (2006) Recording Notch signaling in real time. Dev Neurosci 28:118–127. 10.1159/000090758 16508309 10.1159/000090758

[16] Patel H, Ewels P, Manning J et al (2024) nf-core/rnaseq: nf-core/rnaseq v3.18.0 - Lithium Lynx (3.18.0)

[17] Ewels PA, Peltzer A, Fillinger S et al (2020) The nf-core framework for community-curated bioinformatics pipelines. Nat Biotechnol 38:276–278. 10.1038/s41587-020-0439-x 32055031 10.1038/s41587-020-0439-x

[18] Grüning B, Dale R, Sjödin A et al (2018) Bioconda: sustainable and comprehensive software distribution for the life sciences. Nat Methods 15:475–476. 10.1038/s41592-018-0046-7 29967506 10.1038/s41592-018-0046-7 PMC11070151

[19] da Veiga Leprevost F, Grüning BA, Alves Aflitos S et al (2017) BioContainers: an open-source and community-driven framework for software standardization. Bioinformatics 33:2580–2582. 10.1093/bioinformatics/btx192 28379341 10.1093/bioinformatics/btx192 PMC5870671

[20] Martin M (2011) Cutadapt removes adapter sequences from high-throughput sequencing reads. EMBnet J 17:10. 10.14806/ej.17.1.200

[21] Harrison PW, Amode MR, Austine-Orimoloye O et al (2024) Ensembl 2024. Nucleic Acids Res 52:D891–D899. 10.1093/nar/gkad1049 37953337 10.1093/nar/gkad1049 PMC10767893

[22] Dobin A, Davis CA, Schlesinger F et al (2013) STAR: ultrafast universal RNA-seq aligner. Bioinformatics 29:15–21. 10.1093/bioinformatics/bts635 23104886 10.1093/bioinformatics/bts635 PMC3530905

[23] Patro R, Duggal G, Love MI et al (2017) Salmon provides fast and bias-aware quantification of transcript expression. Nat Methods 14:417–419. 10.1038/nmeth.4197 28263959 10.1038/nmeth.4197 PMC5600148

[24] Love MI, Huber W, Anders S (2014) Moderated Estimation of fold change and dispersion for RNA-seq data with DESeq2. Genome Biol 15:550. 10.1186/s13059-014-0550-8 25516281 10.1186/s13059-014-0550-8 PMC4302049

[25] Yeh BK, Igarashi M, Eliseenkova AV et al (2003) Structural basis by which alternative splicing confers specificity in fibroblast growth factor receptors. P Natl Sci 100:2266–2271. 10.1073/pnas.0436500100 10.1073/pnas.0436500100 PMC151329 12591959

[26] Bednar D, Beerens K, Sebestova E et al (2015) FireProt: Energy- and Evolution-Based computational design of thermostable Multiple-Point mutants. PLoS Comput Biol 11:e1004556. 10.1371/journal.pcbi.1004556 26529612 10.1371/journal.pcbi.1004556 PMC4631455

[27] Guerois R, Nielsen JE, Serrano L (2002) Predicting changes in the stability of proteins and protein complexes: A study of more than 1000 mutations. J Mol Biol 320:369–387. 10.1016/S0022-2836(02)00442-4 12079393 10.1016/S0022-2836(02)00442-4

[28] Kellogg EH, Leaver-Fay A, Baker D (2011) Role of conformational sampling in computing mutation‐induced changes in protein structure and stability. Proteins 79:830–838. 10.1002/prot.22921 21287615 10.1002/prot.22921 PMC3760476

[29] Dvorak P, Bednar D, Vanacek P et al (2018) Computer-assisted engineering of hyperstable fibroblast growth factor 2. Biotechnol Bioeng 115:850–862. 10.1002/bit.26531 29278409 10.1002/bit.26531

[30] Frickey T, Lupas A (2004) CLANS: a Java application for visualizing protein families based on pairwise similarity. Bioinformatics 20:3702–3704. 10.1093/bioinformatics/bth444 15284097 10.1093/bioinformatics/bth444

[31] Edgar RC (2004) MUSCLE: a multiple sequence alignment method with reduced time and space complexity. BMC Bioinformatics 5:113. 10.1186/1471-2105-5-113 15318951 10.1186/1471-2105-5-113 PMC517706

[32] Steipe B, Schiller B, Plückthun A, Steinbacher S (1994) Sequence statistics reliably predict stabilizing mutations in a protein domain. J Mol Biol 240:188–192. 10.1006/jmbi.1994.1434 8028003 10.1006/jmbi.1994.1434

[33] Mistry J, Chuguransky S, Williams L et al (2021) Pfam: the protein families database in 2021. Nucleic Acids Res 49:D412–D419. 10.1093/nar/gkaa913 33125078 10.1093/nar/gkaa913 PMC7779014

[34] Wallace AC, Laskowski RA, Thornton JM (1995) LIGPLOT: a program to generate schematic diagrams of protein-ligand interactions. Protein Eng Des Sel 8:127–134. 10.1093/protein/8.2.127 10.1093/protein/8.2.127 7630882

[35] Pellegrini L, Burke DF, von Delft F et al (2000) Crystal structure of fibroblast growth factor receptor ectodomain bound to ligand and heparin. Nature 407:1029–1034. 10.1038/35039551 11069186 10.1038/35039551

[36] Schlessinger J, Plotnikov AN, Ibrahimi OA et al (2000) Crystal structure of a ternary FGF-FGFR-Heparin complex reveals a dual role for heparin in FGFR binding and dimerization. Mol Cell 6:743–750. 10.1016/S1097-2765(00)00073-3 11030354 10.1016/s1097-2765(00)00073-3

[37] Laskowski RA, Rullmann JAC, MacArthur MW et al (1996) AQUA and PROCHECK-NMR: programs for checking the quality of protein structures solved by NMR. J Biomol NMR 8(4):477–486. 10.1007/BF00228148 9008363 10.1007/BF00228148

[38] Sokolowska-Wedzina A, Borek A, Chudzian J et al (2014) Efficient production and purification of extracellular domain of human FGFR-Fc fusion proteins from Chinese hamster ovary cells. Protein Expr Purif 99:50–57. 10.1016/j.pep.2014.03.012 24727156 10.1016/j.pep.2014.03.012

[39] Stoneman MR, Biener G, Ward RJ et al (2019) A general method to quantify ligand-driven oligomerization from fluorescence-based images. Nat Methods 16:493–496. 10.1038/s41592-019-0408-9 31110281 10.1038/s41592-019-0408-9 PMC7617210

[40] Zapata-Mercado E, Biener G, McKenzie DM et al (2022) The efficacy of receptor tyrosine kinase EphA2 autophosphorylation increases with EphA2 oligomer size. J Biol Chem 298:102370. 10.1016/j.jbc.2022.102370 35970390 10.1016/j.jbc.2022.102370 PMC9512837

[41] Yu J, Hu K, Smuga-Otto K et al (2009) Human induced pluripotent stem cells free of vector and transgene sequences. Sci (1979) 324:797–801. 10.1126/science.1172482 10.1126/science.1172482 PMC2758053 19325077

[42] Miller AJ, Dye BR, Ferrer-Torres D et al (2019) Generation of lung organoids from human pluripotent stem cells in vitro. Nat Protoc 14:518–540. 10.1038/s41596-018-0104-8 30664680 10.1038/s41596-018-0104-8 PMC6531049

[43] Dye BR, Hill DR, Ferguson MA et al (2015) In vitro generation of human pluripotent stem cell derived lung organoids. Elife 4:e05098. 10.7554/eLife.05098 25803487 10.7554/eLife.05098 PMC4370217

[44] Jose SS, De Zuani M, Tidu F et al (2020) Comparison of two human organoid models of lung and intestinal inflammation reveals Toll-like receptor signalling activation and monocyte recruitment. Clin Transl Immunol 9(5):e1131. 10.1002/cti2.1131 10.1002/cti2.1131 PMC7200218 32377340

[45] Seimetz M, Sommer N, Bednorz M et al (2020) NADPH oxidase subunit NOXO1 is a target for emphysema treatment in COPD. Nat Metab 2:532–546. 10.1038/s42255-020-0215-8 32694733 10.1038/s42255-020-0215-8

[46] Wu C-Y, Cilic A, Pak O et al (2023) CEACAM6 as a novel therapeutic target to boost HO-1—mediated antioxidant defense in COPD. Am J Respir Crit Care Med 207:1576–1590. 10.1164/rccm.202208-1603OC 37219322 10.1164/rccm.202208-1603OC

[47] Alsafadi HN, Staab-Weijnitz CA, Lehmann M et al (2017) An ex vivo model to induce early fibrosis-like changes in human precision-cut lung slices. Am J Physiol-Lung C 312:L896–L902. 10.1152/ajplung.00084.2017 10.1152/ajplung.00084.2017 28314802

[48] Van Dijk EM, Culha S, Menzen MH et al (2017) Elastase-Induced parenchymal disruption and airway hyper responsiveness in mouse precision cut lung slices: toward an ex vivo COPD model. Front Physiol 7:657. 10.3389/fphys.2016.00657 28101062 10.3389/fphys.2016.00657 PMC5209351

[49] Hadzic S, Wu C-Y, Gredic M et al (2023) Fibroblast growth factor 10 reverses cigarette smoke- and elastase-induced emphysema and pulmonary hypertension in mice. Eur Respir J 62:2201606. 10.1183/13993003.01606-2022 37884305 10.1183/13993003.01606-2022 PMC10632559

[50] Ornitz DM, Xu J, Colvin JS et al (1996) Receptor specificity of the fibroblast growth factor family. J Biol Chem 271:15292–15297. 10.1074/jbc.271.25.15292 8663044 10.1074/jbc.271.25.15292

[51] Ibrahimi OA, Zhang F, Eliseenkova AV et al (2004) Biochemical analysis of pathogenic ligand-dependent FGFR2 mutations suggests distinct pathophysiological mechanisms for craniofacial and limb abnormalities. Hum Mol Genet 13:2313–2324. 10.1093/hmg/ddh235 15282208 10.1093/hmg/ddh235 PMC4140565

[52] Hadari YR, Gotoh N, Kouhara H et al (2001) Critical role for the docking-protein FRS2α in FGF receptor-mediated signal transduction pathways. P Natl Sci 98:8578–8583. 10.1073/pnas.161259898 10.1073/pnas.161259898 PMC37478 11447289

[53] Fafilek B, Balek L, Bosakova MK et al (2018) The inositol phosphatase SHIP2 enables sustained ERK activation downstream of FGF receptors by recruiting Src kinases. Sci Signal 11(548):eaap8608. 10.1126/scisignal.aap8608 30228226 10.1126/scisignal.aap8608 PMC12677889

[54] Al Alam D, Danopoulos S, Schall K et al (2015) Fibroblast growth factor 10 alters the balance between goblet and Paneth cells in the adult mouse small intestine. Am J Physiol-Gastr L 308:G678–G690. 10.1152/ajpgi.00158.2014 10.1152/ajpgi.00158.2014 PMC4398841 25721301

[55] Herriges JC, Verheyden JM, Zhang Z et al (2015) FGF-Regulated ETV transcription factors control FGF-SHH feedback loop in lung branching. Dev Cell 35:322–332. 10.1016/j.devcel.2015.10.006 26555052 10.1016/j.devcel.2015.10.006 PMC4763945

[56] Morgani SM, Saiz N, Garg V et al (2018) A Sprouty4 reporter to monitor FGF/ERK signaling activity in ESCs and mice. Dev Biol 441:104–126. 10.1016/j.ydbio.2018.06.017 29964027 10.1016/j.ydbio.2018.06.017 PMC6455974

[57] Minowada G, Jarvis LA, Chi CL et al (1999) Vertebrate sprouty genes are induced by FGF signaling and can cause chondrodysplasia when overexpressed. Development 126:4465–4475. 10.1242/dev.126.20.4465 10498682 10.1242/dev.126.20.4465

[58] Li C, Scott DA, Hatch E et al (2007) Dusp6 (Mkp3) is a negative feedback regulator of FGF-stimulated ERK signaling during mouse development. Development 134:167–176. 10.1242/dev.02701 17164422 10.1242/dev.02701 PMC2424197

[59] Zhang X, Ibrahimi OA, Olsen SK et al (2006) Receptor specificity of the fibroblast growth factor family. J Biol Chem 281:15694–15700. 10.1074/jbc.M601252200 16597617 10.1074/jbc.M601252200 PMC2080618

[60] Krejci P, Salazar L, Goodridge HS et al (2008) STAT1 and STAT3 do not participate in FGF-mediated growth arrest in chondrocytes. J Cell Sci 121:272–281. 10.1242/jcs.017160 18198189 10.1242/jcs.017160

[61] Krejci P, Prochazkova J, Bryja V et al (2009) Fibroblast growth factor inhibits interferon γ-STAT1 and Interleukin 6-STAT3 signaling in chondrocytes. Cell Signal 21:151–160. 10.1016/j.cellsig.2008.10.006 18950705 10.1016/j.cellsig.2008.10.006 PMC2655766

[62] Raucci A, Laplantine E, Mansukhani A, Basilico C (2004) Activation of the ERK1/2 and p38 Mitogen-activated protein kinase pathways mediates fibroblast growth Factor-induced growth arrest of chondrocytes. J Biol Chem 279:1747–1756. 10.1074/jbc.M310384200 14593093 10.1074/jbc.M310384200

[63] Kolupaeva V, Basilico C (2012) Overexpression of Cyclin E/CDK2 complexes overcomes FGF-induced cell cycle arrest in the presence of hypophosphorylated Rb proteins. Cell Cycle 11:2557–2566. 10.4161/cc.20944 22713240 10.4161/cc.20944 PMC3404882

[64] Zlinska V, Feketova Z, Czyrek A et al (2025) Specific Inhibition of fibroblast growth factor receptor 1 signaling by a DNA aptamer. Mol Ther Nucleic Acids 36:102405. 10.1016/j.omtn.2024.102405 39759879 10.1016/j.omtn.2024.102405 PMC11700292

[65] Stoneman MR, Raicu N, Biener G, Raicu V (2020) Fluorescence-based methods for the study of Protein-Protein interactions modulated by ligand binding. Curr Pharm Des 26:5668–5683. 10.2174/1381612826666201116120934 33200695 10.2174/1381612826666201116120934

[66] Paul MD, Grubb HN, Hristova K (2020) Quantifying the strength of heterointeractions among receptor tyrosine kinases from different subfamilies: implications for cell signaling. J Biol Chem 295:9917–9933. 10.1074/jbc.RA120.013639 32467228 10.1074/jbc.RA120.013639 PMC7380177

[67] Paul MD, Rainwater R, Zuo Y et al (2021) Probing membrane protein association using Concentration-Dependent number and brightness. Angew Chem Int Edit 60:6503–6508. 10.1002/anie.202010049 10.1002/anie.202010049 PMC7940563 33351993

[68] Singh DR, Ahmed F, Sarabipour S, Hristova K (2017) Intracellular domain contacts contribute to Ecadherin constitutive dimerization in the plasma membrane. J Mol Biol 429:2231–2245. 10.1016/j.jmb.2017.05.020 28549925 10.1016/j.jmb.2017.05.020 PMC5528180

[69] Karl K, Del Piccolo N, Light T et al (2024) Ligand bias underlies differential signaling of multiple FGFs via FGFR1. Elife 12:RP88144. 10.7554/eLife.88144 38568193 10.7554/eLife.88144 PMC10990489

[70] Chioni A-M, Grose R (2009) Negative regulation of fibroblast growth factor 10 (FGF-10) by polyoma enhancer activator 3 (PEA3). Eur J Cell Biol 88:371–384. 10.1016/j.ejcb.2009.01.004 19410332 10.1016/j.ejcb.2009.01.004 PMC2691923

[71] Veth TS, Francavilla C, Heck AJR, Altelaar M (2023) Elucidating fibroblast growth Factor–Induced Kinome dynamics using targeted mass spectrometry and dynamic modeling. Mol Cell Proteom 22:100594. 10.1016/j.mcpro.2023.100594 10.1016/j.mcpro.2023.100594 PMC10368922 37328066

[72] Kharitonenkov A, Shiyanova TL, Koester A et al (2005) FGF-21 as a novel metabolic regulator. J Clin Invest 115:1627–1635. 10.1172/JCI23606 15902306 10.1172/JCI23606 PMC1088017

[73] Geer DJ, Swartz DD, Andreadis ST (2005) Biomimetic delivery of keratinocyte growth factor upon cellular demand for accelerated wound healing in vitro and in vivo. Am J Pathol 167:1575–1586. 10.1016/S0002-9440(10)61242-4 16314471 10.1016/S0002-9440(10)61242-4 PMC1613189

[74] Szymczyk J, Czyrek A, Otlewski J, Zakrzewska M (2023) FGF1 protects MCF-7 cells against Taltobulin through both the MEKs/ERKs and PI3K/AKT signaling pathway. Biomedicines 11:1856. 10.3390/biomedicines11071856 37509496 10.3390/biomedicines11071856 PMC10376943

[75] Volckaert T, Campbell A, Dill E et al (2013) Localized Fgf10 expression is not required for lung branching morphogenesis but prevents differentiation of epithelial progenitors. Development 140:3731–3742. 10.1242/dev.096560 23924632 10.1242/dev.096560 PMC3754473

[76] Abler LL, Mansour SL, Sun X (2009) Conditional gene inactivation reveals roles for *Fgf10* and *Fgfr2* in Establishing a normal pattern of epithelial branching in the mouse lung. Dev Dynam 238:1999–2013. 10.1002/dvdy.22032 10.1002/dvdy.22032 PMC3538083 19618463

[77] Hashimoto S, Nakano H, Suguta Y et al (2012) Exogenous fibroblast growth Factor-10 induces cystic lung development with altered target gene expression in the presence of heparin in cultures of embryonic rat lung. Pathobiology 79:127–143. 10.1159/000334839 22261751 10.1159/000334839 PMC3290038

[78] Ohuchi H, Nakagawa T, Yamamoto A et al (1997) The mesenchymal factor, FGF10, initiates and maintains the outgrowth of the chick limb bud through interaction with FGF8, an apical ectodermal factor. Development 124:2235–2244. 10.1242/dev.124.11.2235 9187149 10.1242/dev.124.11.2235

[79] Dubey VK, Lee J, Somasundaram T et al (2007) Spackling the crack: stabilizing human fibroblast growth Factor-1 by targeting the N and C terminus β-Strand interactions. J Mol Biol 371:256–268. 10.1016/j.jmb.2007.05.065 17570396 10.1016/j.jmb.2007.05.065

[80] Jan Vilim, Ghazalova T, Petulova E et al (2023) Computer-assisted stabilization of fibroblast growth factor FGF-18. Comput Struct Biotechnol J 21:5144–5152. 10.1016/j.csbj.2023.10.009 37920818 10.1016/j.csbj.2023.10.009 PMC10618113

[81] Sugawara S, Ito T, Sato S et al (2014) Production of an aminoterminally truncated, stable type of bioactive mouse fibroblast growth factor 4 in Escherichia coli. J Biosci Bioeng 117:525–530. 10.1016/j.jbiosc.2013.10.009 24210555 10.1016/j.jbiosc.2013.10.009

[82] Qin K, Yu M, Fan J et al (2024) Canonical and noncanonical Wnt signaling: multilayered mediators, signaling mechanisms and major signaling crosstalk. Genes Dis 11:103–134. 10.1016/j.gendis.2023.01.030 37588235 10.1016/j.gendis.2023.01.030 PMC10425814

[83] Wu M, Wu S, Chen W, Li Y-P (2024) The roles and regulatory mechanisms of TGF-β and BMP signaling in bone and cartilage development, homeostasis and disease. Cell Res 34:101–123. 10.1038/s41422-023-00918-9 38267638 10.1038/s41422-023-00918-9 PMC10837209

[84] Martinez-Hackert E, Sundan A, Holien T (2021) Receptor binding competition: A paradigm for regulating TGF-β family action. Cytokine Growth Factor Rev 57:39–54. 10.1016/j.cytogfr.2020.09.003 33087301 10.1016/j.cytogfr.2020.09.003 PMC7897244

[85] Cruciat C-M, Niehrs C (2013) Secreted and transmembrane Wnt inhibitors and activators. Cold Spring Harb Perspect Biol 5:a015081–a015081. 10.1101/cshperspect.a015081 23085770 10.1101/cshperspect.a015081 PMC3578365

[86] Correns A, Zimmermann L-MA, Baldock C, Sengle G (2021) BMP antagonists in tissue development and disease. Matrix Biol Plus 11:100071. 10.1016/j.mbplus.2021.100071 34435185 10.1016/j.mbplus.2021.100071 PMC8377005

[87] Lee H, Camuto CM, Niehrs C (2024) R-Spondin 2 governs xenopus left-right body axis formation by Establishing an FGF signaling gradient. Nat Commun 15:1003. 10.1038/s41467-024-44951-7 38307837 10.1038/s41467-024-44951-7 PMC10837206

[88] Derrick T, Grillo AO, Vitharana SN et al (2007) Effect of polyanions on the structure and stability of repifermin™ (Keratinocyte growth Factor-2). J Pharm Sci 96:761–776. 10.1002/jps.20797 17094125 10.1002/jps.20797

[89] Govind Kumar V, Agrawal S, Kumar TKS, Moradi M (2021) Mechanistic picture for monomeric human fibroblast growth factor 1 stabilization by heparin binding. J Phys Chem B 125:12690–12697. 10.1021/acs.jpcb.1c07772 34762427 10.1021/acs.jpcb.1c07772

[90] Bellusci S, Grindley J, Emoto H et al (1997) Fibroblast growth factor 10 (FGF10) and branching morphogenesis in the embryonic mouse lung. Development 124:4867–4878. 10.1242/dev.124.23.4867 9428423 10.1242/dev.124.23.4867

[91] Yin Y, Wang F, Ornitz DM (2011) Mesothelial- and epithelial-derived FGF9 have distinct functions in the regulation of lung development. Development 138:3169–3177. 10.1242/dev.065110 21750028 10.1242/dev.065110 PMC3188607

[92] Yin Y, Ornitz DM (2020) FGF9 and FGF10 activate distinct signaling pathways to direct lung epithelial specification and branching. Sci Signal 13(621):eaay4353. 10.1126/scisignal.aay4353 32127497 10.1126/scisignal.aay4353 PMC7271816

[93] Xu X, Weinstein M, Li C et al (1998) Fibroblast growth factor receptor 2 (FGFR2)-mediated reciprocal regulation loop between FGF8 and FGF10 is essential for limb induction. Development 125:753–765. 10.1242/dev.125.4.753 9435295 10.1242/dev.125.4.753

[94] Sun X, Mariani FV, Martin GR (2002) Functions of FGF signalling from the apical ectodermal ridge in limb development. Nature 418:501–508. 10.1038/nature00902 12152071 10.1038/nature00902

[95] Gros J, Tabin CJ (2014) Vertebrate limb bud formation is initiated by localized Epithelial-to-Mesenchymal transition. Sci (1979) 343:1253–1256. 10.1126/science.1248228 10.1126/science.1248228 PMC4097009 24626928

[96] Havens BA, Rodgers B, Mina M (2006) Tissue-specific expression of Fgfr2b and Fgfr2c isoforms, Fgf10 and Fgf9 in the developing chick mandible. Arch Oral Biol 51:134–145. 10.1016/j.archoralbio.2005.06.011 16105644 10.1016/j.archoralbio.2005.06.011

[97] Bell GW, Yatskievych TA, Antin PB (2004) GEISHA, a whole-mount in situ hybridization gene expression screen in chicken embryos. Dev Dynam 229:677–687. 10.1002/dvdy.10503 10.1002/dvdy.10503 14991723

[98] Krejci P, Krakow D, Mekikian PB, Wilcox WR (2007) Fibroblast growth factors 1, 2, 17, and 19 are the predominant FGF ligands expressed in human fetal growth plate cartilage. Pediatr Res 61:267–272. 10.1203/pdr.0b013e318030d157 17314681 10.1203/pdr.0b013e318030d157

[99] Delezoide A-L, Benoist-Lasselin C, Legeai-Mallet L et al (1998) Spatio-temporal expression of FGFR 1, 2 and 3 genes during human embryo-fetal ossification. Mech Dev 77:19–30. 10.1016/S0925-4773(98)00133-6 9784595 10.1016/s0925-4773(98)00133-6

[100] Olney RC, Wang J, Sylvester JE, Mougey EB (2004) Growth factor regulation of human growth plate chondrocyte proliferation in vitro. Biochem Biophys Res Commun 317:1171–1182. 10.1016/j.bbrc.2004.03.170 15094393 10.1016/j.bbrc.2004.03.170

[101] Yoshioka H, Kagawa K, Minamizaki T et al (2023) Developmental impairments of craniofacial bone and cartilage in Transgenic mice expressing FGF10. Bone Rep 18:101692. 10.1016/j.bonr.2023.101692 37275784 10.1016/j.bonr.2023.101692 PMC10236464

[102] Alexandre P, Wassef M (2005) Does the isthmic organizer influence D/V patterning of the midbrain? Brain Res Rev 49:127–133. 10.1016/j.brainresrev.2005.04.003 15951023 10.1016/j.brainresrev.2005.04.003

