# Supplementary material for: Increased thermal stability of FGF10 leads to ectopic signaling during development
Source: Cell Mol Life Sci. 2025 Apr 21;82(1):167. doi: 10.1007/s00018-025-05681-1 (PMC12011707; doi:10.1007/s00018-025-05681-1)
Supplement: Supplementary file 1 — Supplementary Material 1 [file 18_2025_5681_MOESM1_ESM.pdf]

Supplementary information

Increased thermal stability of FGF10 leads to ectopic signaling during development

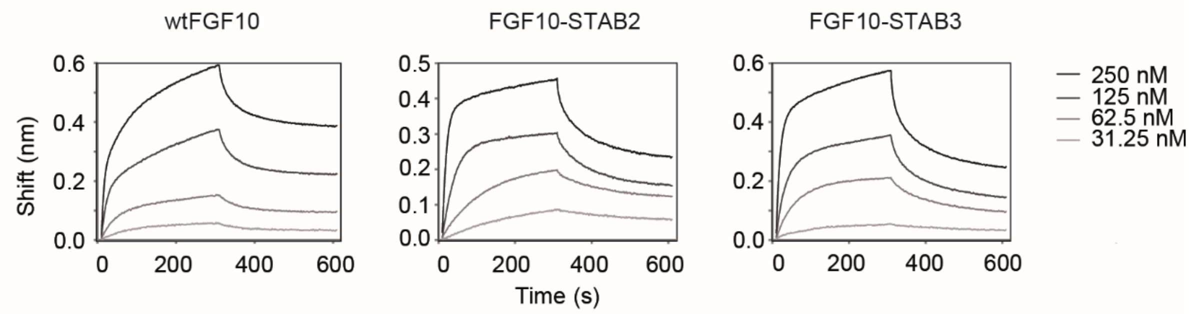

Protein	*K_D [nM]*
wtFGF10	764 ± 132
FGF10-STAB2	996 ± 380
FGF10-STAB3	913 ± 449

Figure S1 FGFR2b interaction kinetics of wildtype FGF10, FGF10-STAB2 and FGF10-STAB3 analyzed by biolayer interferometry. Recombinant extracellular domain of FGFR2b fused to the Fc fragment was immobilized on a Protein A biosensor. Dissociation constants were calculated from fitted saturation binding curves. Representative sensograms for three (n) independent experiments are shown.

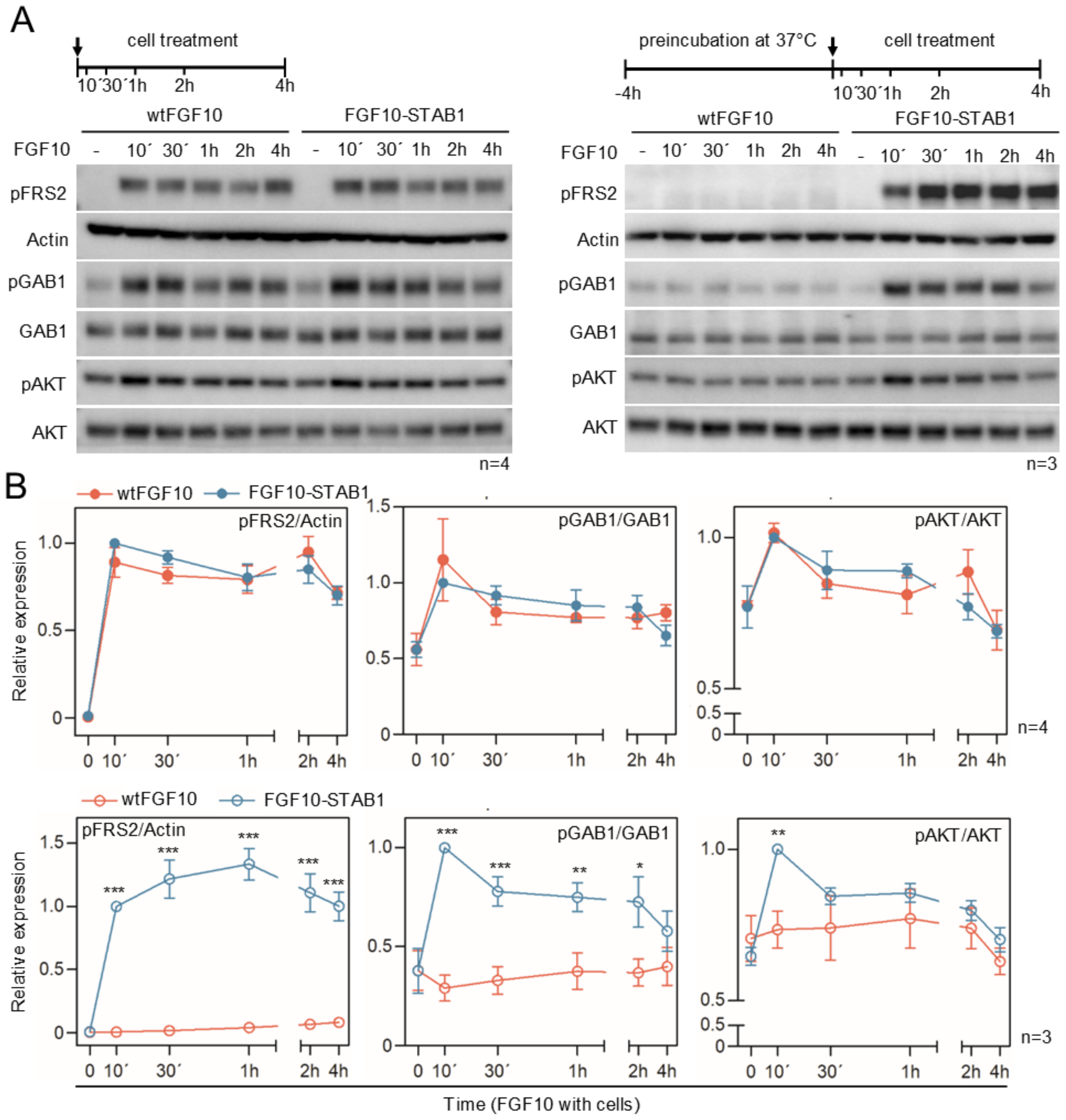

Figure S2 (**A**) 4MBr-5 cells were serum-starved for two days, treated with fresh or preincubated (4h/37°C) FGF10 for indicated times, and the FGF10-mediated phosphorylation (p) of FRS2, GAB1 and AKT was analyzed by western blot. Actin, total GAB1 and total AKT serve as loading controls. (**B**) Phosphorylation signals were quantified and graphed (mean±SEM, data relative to the 10 minutes FGF10-STAB1; n, number of independent experiments; Welch´s *t*-test, ***p<0.001, **p<0.01, *p<0.05).

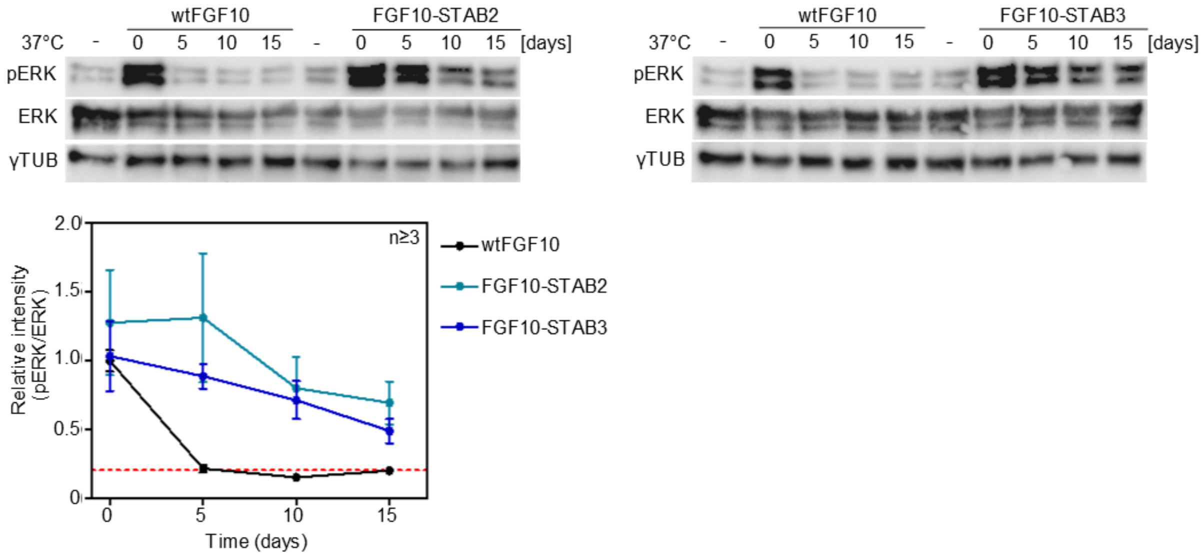

Figure S3 Recombinant FGF10 (0.5 μg/ml) was incubated at 37°C in DMEM supplemented with 10% FBS for 5-15 days. MCF7 cells were stimulated with preincubated FGF10 (20 ng/ml) for 15 minutes and analyzed for activation of ERK pathway. Data are representative for at least three independent experiments (n). pERK signal was quantified and normalized to total ERK (mean±SD for at least three independent experiments).

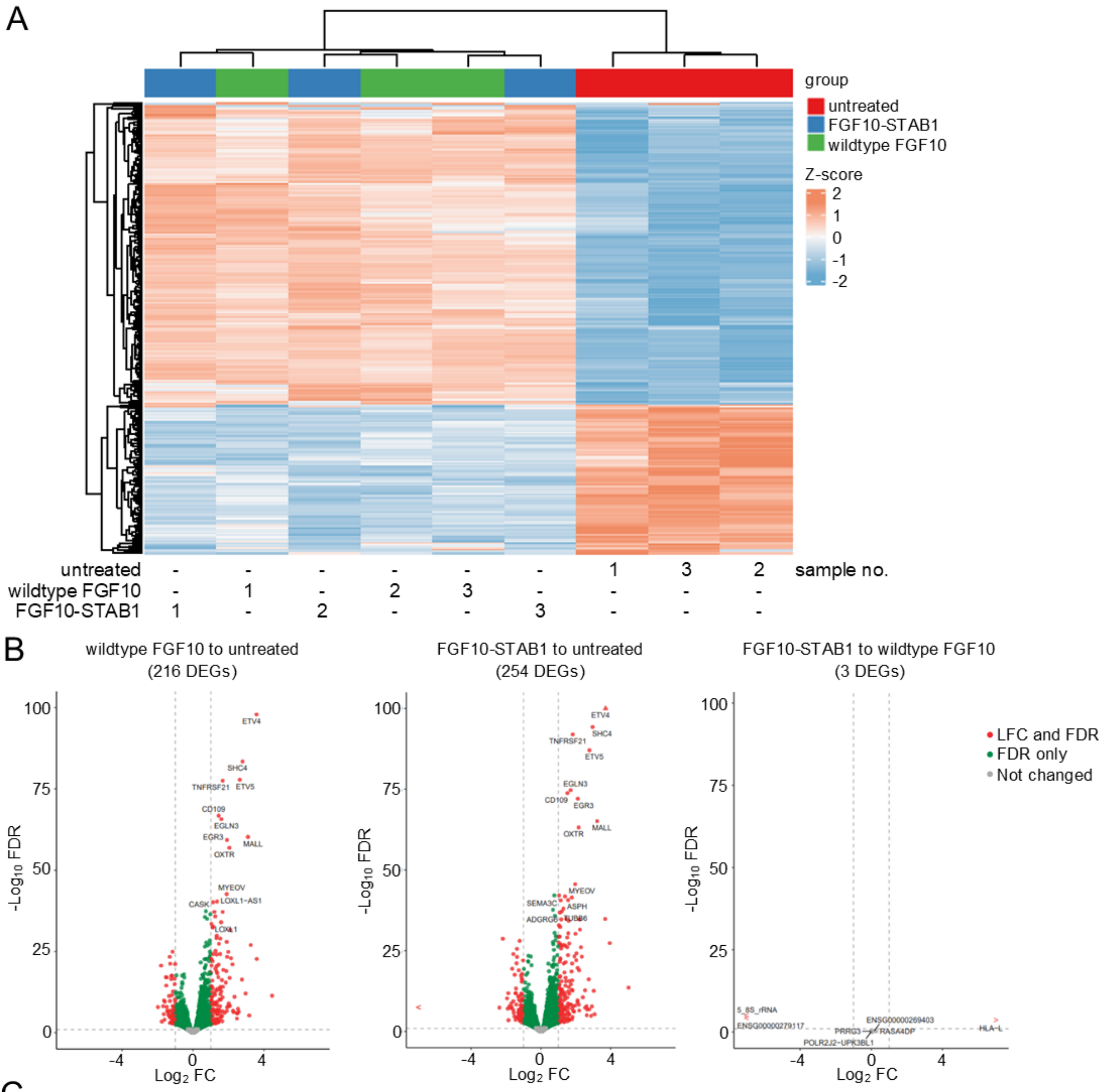

C

Symbol	*Log2 FGF10-STAB1 to wildtype FGF10*	*Description*
HLA-L	7.10	major histocompatibility complex, class I, L (pseudogene)
ENSG00000279117	-21.9	non-coding sequence (TEC; to be experimentaly confirmed)
5_8S_rRNA	-23.4	5.8S ribosomal RNA (pseudogene)

Figure S4 Differential expression analysis between MCF7 cells treated with wildtype FGF10 or FGF10-STAB1 and untreated cells. MCF7 cells were serum starved for two days, treated with wildtype FGF10 or FGF10-STAB1 (20 ng/ml) for 8 hours, and analyzed for changes in gene expression. Three independent cell samples were analyzed in each group. Compared to untreated controls, wildtype FGF10 regulated expression of 216 genes (175 protein-coding genes), while FGF10-STAB1 modulated the expression of 254 genes (201 protein-coding genes). (**A**) Expression heatmap of genes differentially expressed in at least one of the comparisons shows the minimal difference in induced gene expression changes between wildtype FGF10 and FGF10-STAB1. (**B**) Results of differential expression analysis for individual genes (points) showed as volcano plot. DEG, differentially expressed gene; LFC, log_2 fold changes; FDR, false discovery rate. (**C**) Only three non-coding transcripts were found differentially expressed between FGF10-STAB1 and wildtype FGF10.

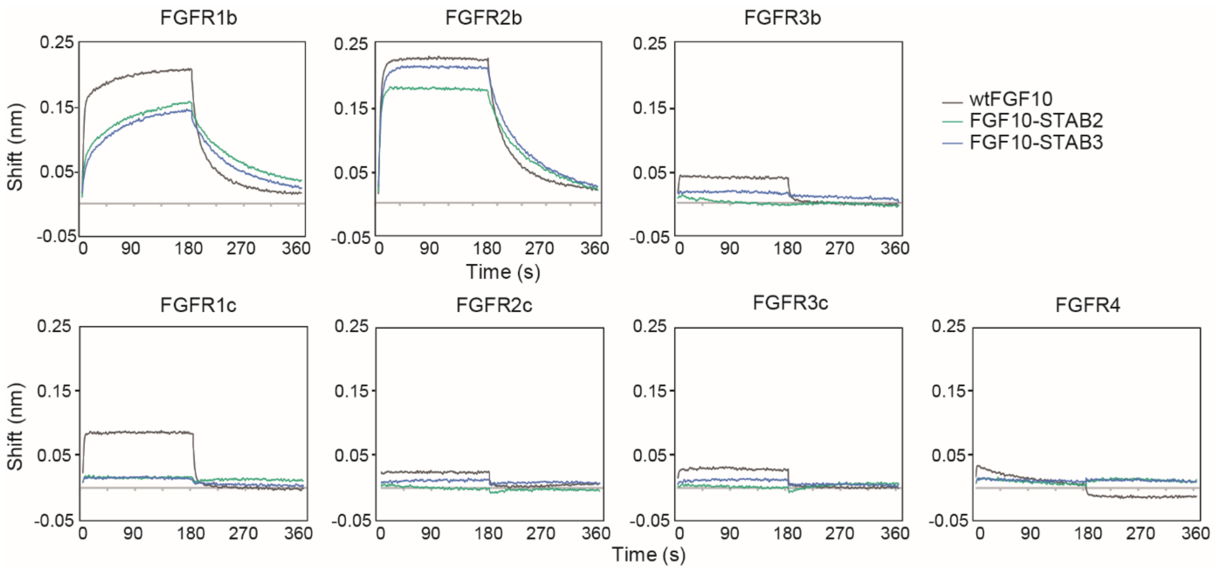

Figure S5 FGFR specificity analysis of wild type of FGF10, FGF10-STAB2 and FGF10-STAB3 variants designed by semi-rational approach assessed by biolayer interferometry (BLI) technique. BLI experiments were performed on an Octet K2 instrument (Sartorius AG) using Protein A biosensors. Recombinant extracellular domains of individual FGFRs (isoforms IIIb and IIIc) fused to the Fc fragment were immobilized on a Protein A biosensor. FGFR coupled to the sensor was then used to capture the analyzed FGF10 variants at a concentration of 250 nM. Association and dissociation processes were monitored for 180 seconds each. ForteBio's Data Analysis 11.0 software was used to analyze the binding curves. Representative sensograms are shown (n=3).

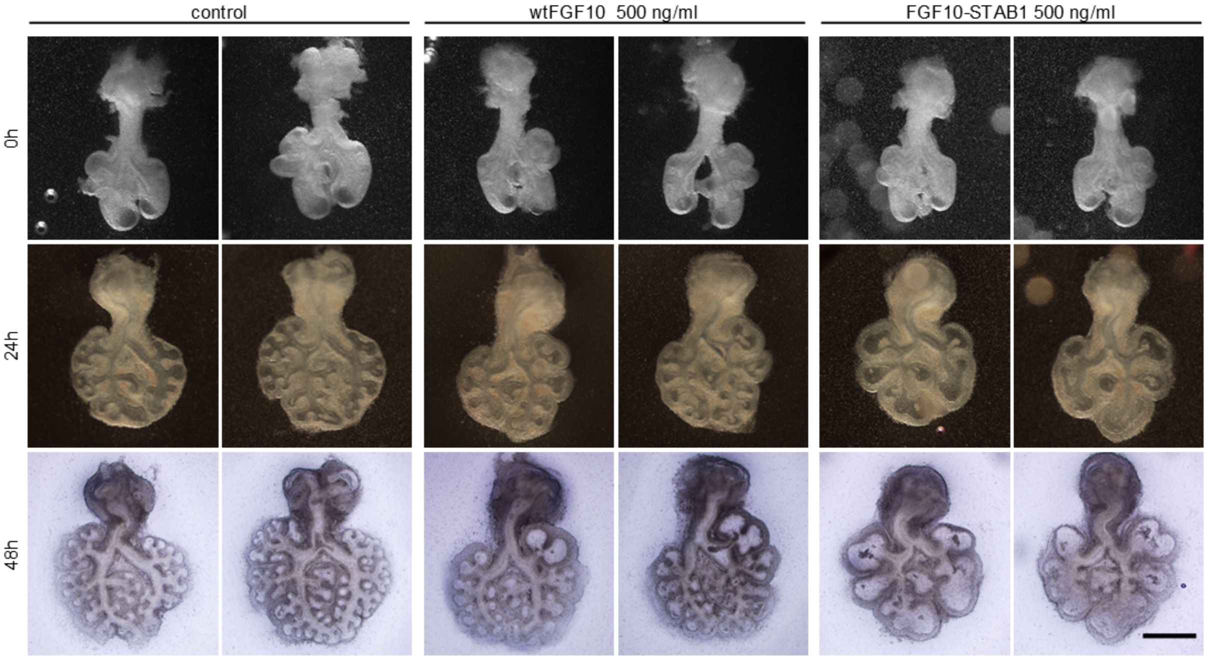

Figure S6 FGF10-STAB1 alters cell differentiation in embryonic lung explants. E11.25 mouse lungs were explanted and incubated for up to 48 hours with 500 ng/ml FGF10 added every 12-24 hours. Overall morphology of lung explants reveals significant suppression of branching by FGF10-STAB1, while only small effect was found with 500 ng/ml of wildtype FGF10 (scale bar, 0.5 mm). Two explants are shown for each treatment.

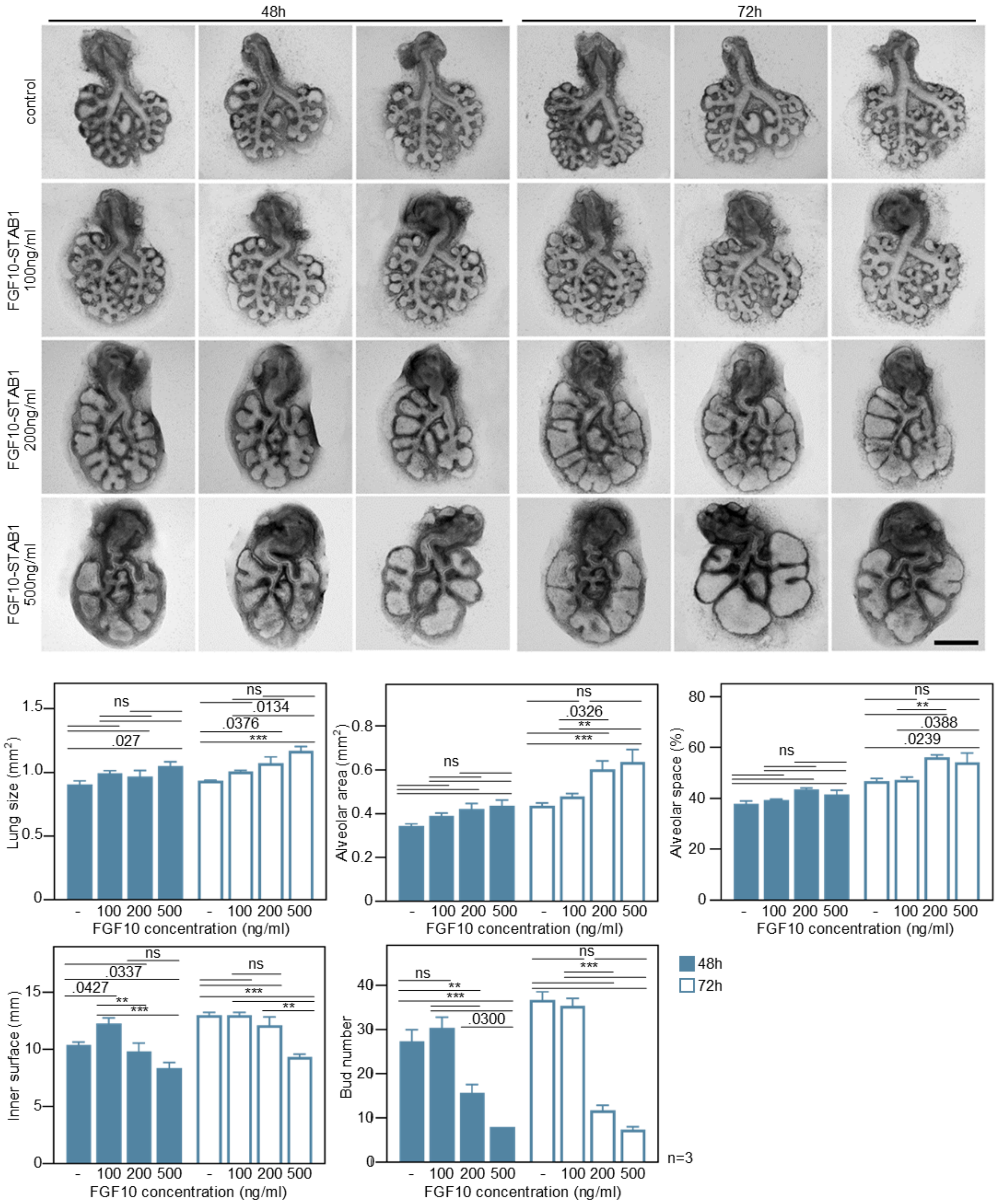

Figure S7 FGF10-STAB1 alters cell differentiation in embryonic lung explants. E11.25 mouse lungs were explanted and incubated for up to 48 and 72 hours with FGF10 added every 12-24 hours. The overall morphology of lung explants reveals significant suppression of branching by FGF10-STAB1, apparent at 200 and 500 ng/ml (scale bar, 0.5 mm). Three explants are shown for each treatment (mean±SEM, Two-Way ANOVA, ***$p<0.001$, **$p<0.01$).

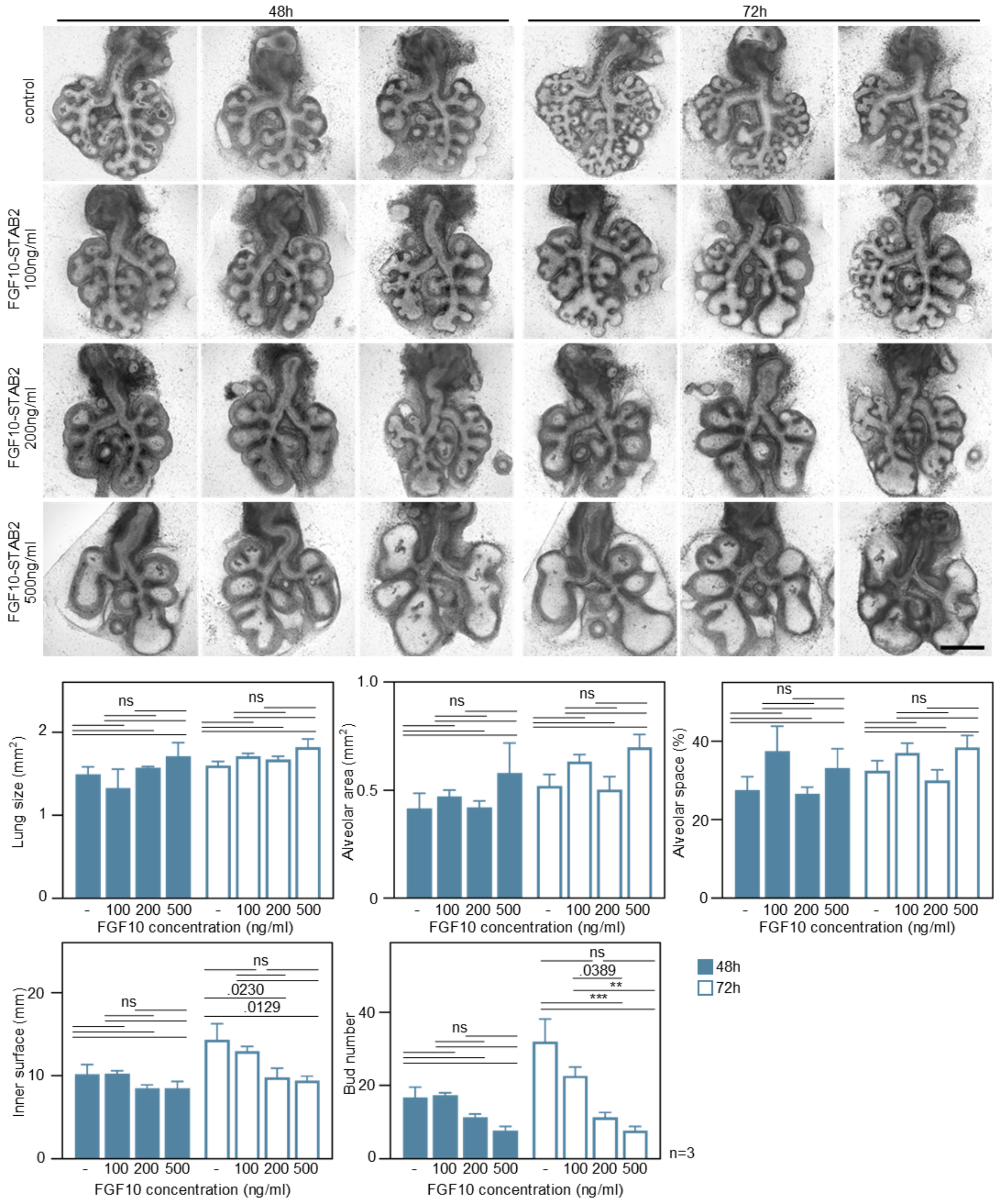

Figure S8 FGF10-STAB2 alters cell differentiation in embryonic lung explants. E11.25 mouse lungs were explanted and incubated for up to 48 and 72 hours with FGF10 added every 12-24 hours. The overall morphology of lung explants reveals significant suppression of branching by FGF10-STAB2, apparent at 100, 200 and 500 ng/ml (scale bar, 0.5 mm). Three explants are shown for each treatment treatment (mean±SEM, Two-Way ANOVA, ***p<0.001, **p<0.01).

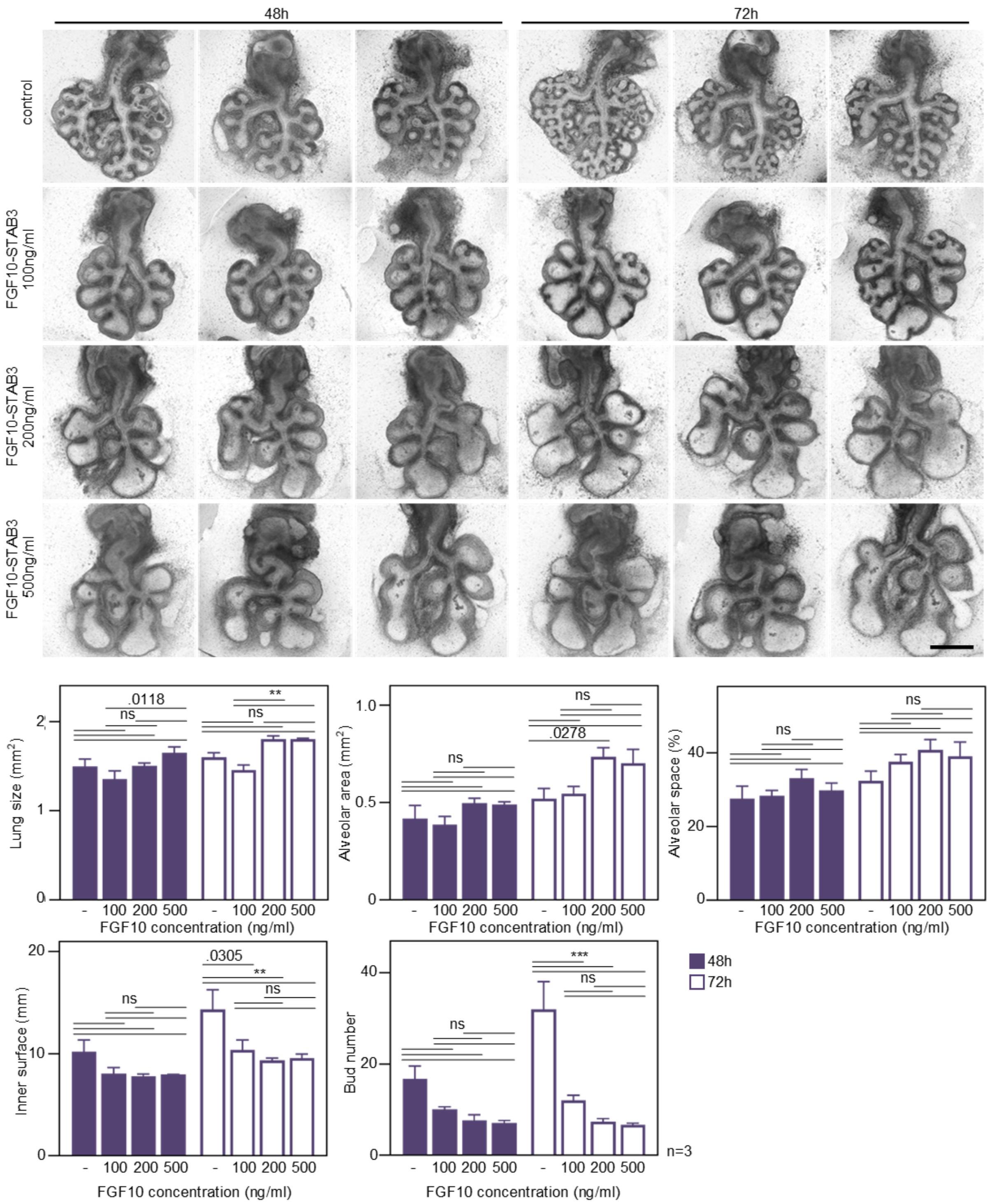

Figure S9 FGF10-STAB3 alters cell differentiation in embryonic lung explants. E11.25 mouse lungs were explanted and incubated for up to 48 and 72 hours with FGF10 added every 12-24 hours. The overall morphology of lung explants reveals significant suppression of branching by FGF10-STAB3, apparent at 100, 200 and 500 ng/ml (scale bar, 0.5 mm). Three explants are shown for each treatment treatment (mean±SEM, Two-Way ANOVA, ***p<0.001, **p<0.01).

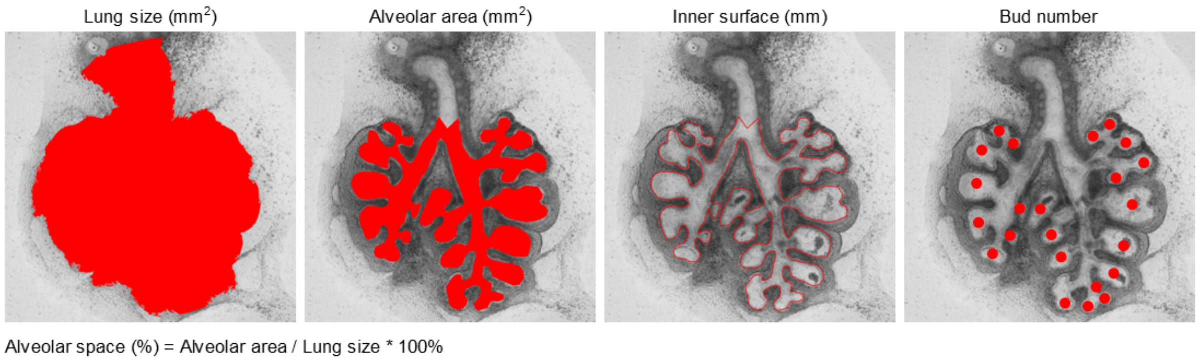

Figure S10 Scheme showing the measurement of embryonic lung explants stimulated with FGF10 variants.

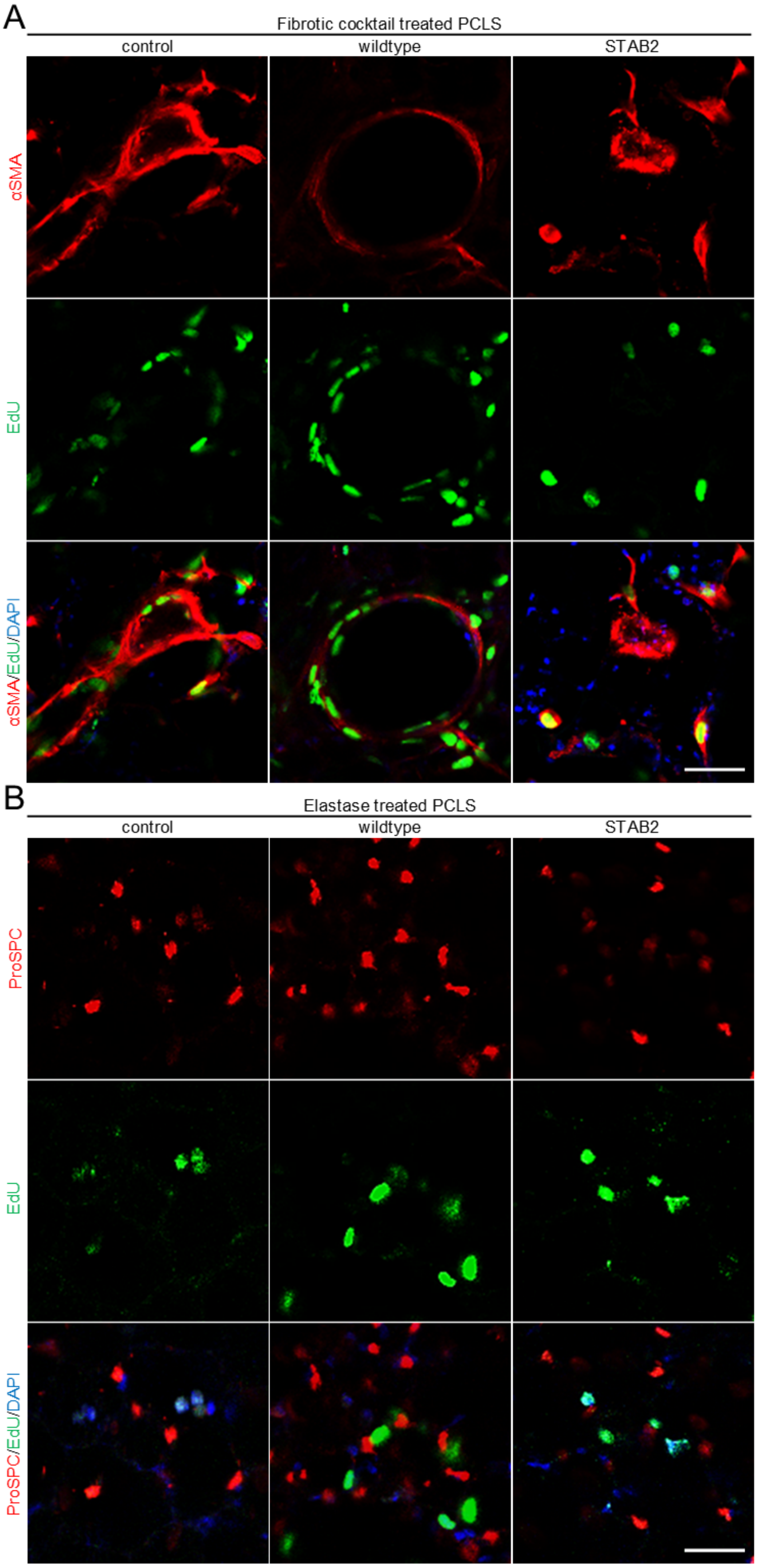

Figure S11 (**A**) Representative confocal images of PCLS fibrosis. PCLS pretreated with

fibrotic cocktail upon 72 hours stimulation with vehicle (control), wildtype FGF10 and FGF-STAB2 (250 ng/ml) were stained with EdU (green), αSMA (red) and DAPI (blue). (**B**) Representative confocal images of PCLS regeneration. PCLS injury was induced by preincubation in elastase for 24 hours before treatment with vehicle, wildtype FGF10 and STAB2 (250 ng/ml) and stained with EdU (green), ProSPC (red) and DAPI (blue). Scale bar 50 µm.

Table S1 Antibodies used in the study. WB, western blot; IHC, immunohistochemistry; 2nd, secondary antibody.

Protein	*Source*	*Cat. No.*	*Method*
Actin	Cell Signaling	3700	WB
AKT	Cell Signaling	4691	WB
$pAKT^{S473}$	Cell Signaling	4060	WB
ERK	Cell Signaling	9102	WB
$pERK^{T202/Y204}$	Cell Signaling	9101	WB
$pERK^{T202/Y204}$	Cell Signaling	4376	WB
FGF10	Santa Cruz	sc-7917	WB
$pFRS2^{Y196}$	Cell Signaling	3864	WB
$pFRS2^{Y436}$	Cell Signaling	3861	WB
Gab1	Cell Signaling	3232	WB
$pGab1^{Y627}$	Cell Signaling	3231	WB
γTubulin	Sigma Aldrich	T6557	WB
V5	Invitrogen	46-0705	WB
mouse IgG	Sigma Aldrich	A6782	2nd, WB
rabbit IgG	Sigma Aldrich	A6667	2nd, WB
mouse IgG	Jackson ImmunoResearch	115-035-003	2nd, WB
rabbit IgG	Jackson ImmunoResearch	111-035-144	2nd, WB
αSMA	Abcam	ab5694	IHC
αSMA	Merck Millipore	C6198	IHC
DRAQ5	Thermo Fisher	62251	IHC
E-cadherin	eBioscience	53-3249-82	IHC
Pro-SPC	Abcam	ab270521	IHC
Sox2	Cell Signaling	2748S	IHC
Sox9	Abcam	ab185230	IHC
Sox9	Sigma Aldrich	HPA001758	IHC
rabbit IgG	Invitrogen	A-31573	2nd, IHC
rat IgG	Invitrogen	A-21208	2nd, IHC

Table S2 Stabilizing mutations identified in FGF10 by energy-based and evolution-based predictions. Mutations identified by evolution-based approach are highlighted in bold. ΔΔG, predicted change in Gibbs free energy; MSA, multiple sequence alignment.

Mutation	*ΔΔG (kcal/mol)*	*Conservation*	*Consensus*	*Amino acids in MSA*
N71E	-2.331	5	-	D, G, E, K, Y, N, F, S, P, V, H
T86F	-1.783	7	-	H, N, P, T, Q
I126Y	-3.167	7	-	I, F, M, L, V
N127Y	-2.637	3	-	G, A, E, Y, K, N, F, Q, T, S, H
S143P	-2.089	7	-	K, V, A, E, T, S
H171Y	-1.996	5	-	Q, R, K, H, N, P, E
R174Y	-3.650	6	-	G, E, H, K, R, T, Q
Q175E	-2.102	6	-	Q, A, E, P, H, R
N181D	-2.100	7	-	N, R, G, D, M, S
V205P	-1.377	2	-	P, V, F, I, Q, M, T, A, E, K, N, L
**I118V**	0.008	8	0.69	M, T, Q, I, E, V
**V123I**	-1.590	8	0.83	L, I, V
**L152F**	0.395	9	0.62	L, F
**Y177F**	-0.100	8	0.83	Y, F

Table S3 *In silico* prediction of stabilizing effect of multiple-point FGF10 mutants. ΔΔG, predicted change in Gibbs free energy.

Mutations	*Sum of single-point mutant contribution ΔΔG (kcal/mol)*	*Calculated values for multiple-point mutants ΔΔG (kcal/mol)*
V123I_L152F	-1.2	-4.3
V123I_Q175E	-3.7	-3.3
V123I_N181D	-3.7	-1.8
L152F_ Q175E	-1.7	-2.2
L152F_N181D	-1.7	-0.8
Q175E_N181D	-4.2	-0.5
V123I_L152F_Q175E	-3.3	-5.2
V123I_L152F_N181D	-3.3	-3.3
V123I_Q175E_N181D	-5.8	-2.7
L152F_Q175E_N181D	-3.8	-3.7
V123I_L152F_Q175E_N181D	-5.4	-5.0

Table S4 Thermodynamic parameters of wildtype FGF10 and its single mutants designed by semi-rational approach, determined from thermal denaturation monitored by measuring ellipticity at 228 nm. Data presented are mean values from three independent experiments +/- SD. Variants with higher stability than the wildtype FGF10 are in bold. The mutations used in STAB1 were italicized. In green are variants designed with a homology strategy. In blue is variant designed to lower affinity for heparin.

Protein	ΔT_{den}	T_{den}	ΔH_{den}
wildtype FGF10	-	53.8±0.8	137.7±23.0
F89H	-10.5	43.3±1.0	107.6±22.7
C150S	-9.0	44.8±1.1	123.2±5.4
M176W	-8.2	45.6±1.7	144.2±27.1
G160Y	-7.5	46.3±0.1	167.8±2.4
E93L	-7.3	46.5±0.7	148.5±7.9
C106S	-6.6	47.2±0.2	144.3±0.5
S99D	-5.8	47.9±0.2	153.0±2.5
K91Q	-3.8	50±0.4	154.6±0.9
N148P	-3.6	50.2±4.8	139.6±30.7
K151V	-2.2	51.6±0.6	149.9±9.6
F167R	-2.1	51.7±0.5	144.8±7.5
E93H	-1.5	52.2±0.3	145.0±6.1
K87N	-1.5	52.3±0.0	148.8±6.3
N196G	-1.4	52.4±0.3	152.3±5.5
Y108F	-0.8	53.0±0.2	156.4±3.1
C106D	-0.7	53.1±0.1	158.6±4.4
A185T	-0.4	53.4±0.0	159.3±1.3
N196Q	-0.2	53.6±0.0	159.5±2.7
W169Y	-0.1	53.7±0.1	146.9±1.9
T114I	0.0	53.8±0.3	155.3±10.3
I126V	0.0	53.8±0.6	105.9±14.0
**K103E**	0.3	54.1±0.3	160.3±11.6
**Q170S**	0.4	54.2±1.0	159.4±1.4
**N148S**	0.6	54.4±0.6	151.5±7.1
**A185R**	0.6	54.4±1.2	154.1±9.9
**K191A**	0.7	54.5±0.1	162.4±5.8
**N135D**	1.1	54.9±0.2	150.7±7.7
**N148D**	1.5	55.3±0.1	155.0±11.5
**T86N**	1.9	55.7±0.8	123.8±5.1
V123I	2.6	56.4±0.3	161.1±1.6
L152F	6.6	60.4±0.7	102.5±15.6

Table S5 Thermodynamic parameters of wildtype FGF10 and its multiple mutants designed by semi-rational approach, determined from thermal denaturation monitored by measuring ellipticity at 228 nm (mean from at least three independent experiments ±SD). The mutations used in STAB1 were italicized.

Protein	ΔT_{den}	T_{den}	H_{den}
wildtype FGF10	-	53.8±0.8	137.7±23.0
W79I_K87G_N129G_Q170R_N181P_R187V	9.7	63.5±0.3	125.1±4.7
W79I_F89Y_E93N_*L152F*	11.1	64.9±1.1	166.1±15.1
W79I_E93N_N129G_*L152F*	12.9	66.7±1.1	172.0±10.8
W79I_L111F_*V123I*_N129G_*L152F*	13.5	67.3±4.5	172.7±22.6
W79I_E93N_*V123I*_N129G_*L152F*	15.1	68.9±0.8	148.0±27.3
W79I_F89Y_*V123I*_N129G_*L152F*	15.5	69.3±1.2	168.3±26.2
W79I_*V123I*_N129G_*L152F*_F167A	16.0	69.8±0.3	117.6±22.5
W79I_*V123I*_N129G_*L152F*	16.7	70.5±0.0	182.3±0.4
W79I_K87G_N129G_Q170R_R187E	16.9	70.7±0.4	131.6±14.4
W79I_K87G_F89Y_*V123I*_N129G_R187V	17.5	71.3±0.6	125.6±35.9
W79I_K87G_E93N_*V123I*_N129G_R187V	18.3	72.1±0.2	137.2±24.8
W79I_K87G_N129G_Q170R_R187V	18.5	72.3±0.6	138.2±18.8
W79I_F89Y_E93N_K94P_*V123I*_N129G_*L152F*_F167A_Q170T	18.6	72.4±2.4	123.3±30.1
W79I_K87G_N129G_Q170R_R187V_R188A	18.8	72±1.3	143.3±15.6
W79I_K87G_N129G_Q170R_R187V_R188E	19.5	73.3±0.8	150.2±15.6
W79I_K94P_*V123I*_N129G_*L152F*	19.8	73.6±0.3	162.5±9.5
W79I_K87G_K94P_N129G_Q170R_R187E	20.4	74.2±0.1	142.3±9.3
W79I_E93N_K94P_*V123I*_N129G_*L152F*_ F167A_Q170T	20.5	74.3±0.4	149.4±6.7
W79I_K87G_*V123I*_N129G_*L152F*	20.7	74.5±0.1	179.8±4.5
W79I_K87G_E93D_K94P_N129G_Q170R_R187E	20.9	74.7±1.0	118.0±19.5
W79I_K87G_K94P_N129G_Q170R_R187V	20.9	74.7±0.6	132.8±5.8

Table S6 The affinity of wildtype FGF10, FGF10-STAB2 and FGF10-STAB3 for heparin. Proteins were eluted from a Heparin-Sepharose column by 0.15-1.5M NaCl gradient. Data shown are mean values of the salt concentration at which proteins were eluted, from at least three independent experiments ± SD.

Protein	*NaCl concentration [M]*
wildtype FGF10	1.057±0.04
FGF10-STAB2	0.855±0.04
FGF10-STAB3	0.785±0.04

Table S7 Genes differentially expressed between wildtype FGF10 or FGF10-STAB1 and untreated MCF7 cells. Only protein-coding genes exhibiting absolute log2 fold change value higher or equal to 1.5 in at least one of the comparisons are shown.

Symbol	*Log_2 fold change*		*Description*
	wildtype to untreated	*STAB1 to untreated*	
B3GNT6	4.46	5.01	UDP-GlcNAc:betaGal beta-1,3-N-acetylglucosaminyltransferase 6
MUC2	3.6	3.94	mucin 2, oligomeric mucus/gel-forming
ETV4	3.59	3.7	ETS variant transcription factor 4
SPRY4	3.26	3.67	sprouty RTK signaling antagonist 4
LGALS7B	3.17	2.87	galectin 7B
MALL	3.1	3.22	mal, T cell differentiation protein like
A2M	2.97	3.02	alpha-2-macroglobulin
SHC4	2.79	2.96	SHC adaptor protein 4
ETV5	2.64	2.78	ETS variant transcription factor 5
KRT20	2.59	2.92	keratin 20
GPR3	2.54	2.54	G protein-coupled receptor 3
C10orf67	2.5	2.77	chromosome 10 open reading frame 67
RAB7B	2.48	3.07	RAB7B, member RAS oncogene family
AXL	2.47	2.66	AXL receptor tyrosine kinase
IL2RB	2.34	2.53	interleukin 2 receptor subunit beta
RARRES2	2.26	2.19	retinoic acid receptor responder 2
AREG	2.14	2.29	amphiregulin
FN1	2.1	2.21	fibronectin 1
ZNF365	2.07	2.19	zinc finger protein 365
OXTR	2.05	2.16	oxytocin receptor
DUSP6	1.99	2.24	dual specificity phosphatase 6
CATSPER1	1.99	1.96	cation channel sperm associated 1
KCNF1	1.93	1.92	potassium voltage-gated channel modifier subfamily F member 1
EGR3	1.92	2.11	early growth response 3
PTGER2	1.9	1.98	prostaglandin E receptor 2
MYEOV	1.9	1.96	myeloma overexpressed
RASGEF1A	1.88	2.02	RasGEF domain family member 1A
TNIK	1.85	2.1	TRAF2 and NCK interacting kinase
SYT13	1.79	1.63	synaptotagmin 13
SDR16C5	1.69	1.79	short chain dehydrogenase/reductase family 16C member 5
TNFRSF21	1.68	1.82	TNF receptor superfamily member 21
DUSP5	1.68	1.77	dual specificity phosphatase 5
TMPRSS11E	1.67	1.84	transmembrane serine protease 11E
KRT23	1.67	1.81	keratin 23
ACOX2	1.64	1.87	acyl-CoA oxidase 2
GPR50	1.64	1.6	G protein-coupled receptor 50
TNFRSF11B	1.63	1.75	TNF receptor superfamily member 11b
EGLN3	1.6	1.7	egl-9 family hypoxia inducible factor 3

KRT7	1.59	1.56	keratin 7
LOXL1	1.58	1.59	lysyl oxidase like 1
UPP1	1.56	1.76	uridine phosphorylase 1
EMP1	1.55	1.72	epithelial membrane protein 1
MUC5AC	1.54	2.03	mucin 5AC, oligomeric mucus/gel-forming
TMT1B	1.54	1.57	thiol methyltransferase 1B
LONRF3	1.54	1.54	LON peptidase N-terminal domain and ring finger 3
NIPAL1	1.53	1.62	NIPA like domain containing 1
LOXL2	1.53	1.48	lysyl oxidase like 2
CDC42EP3	1.52	1.62	CDC42 effector protein 3
KRT86	1.49	1.64	keratin 86
SEC14L2	1.49	1.51	SEC14 like lipid binding 2
SERPINA3	1.46	1.51	serpin family A member 3
CAPN8	1.45	1.81	calpain 8
CD109	1.45	1.52	CD109 molecule
DMP1	1.44	1.53	dentin matrix acidic phosphoprotein 1
RNF183	1.42	1.59	ring finger protein 183
ALOXE3	1.39	1.72	arachidonate lipoxygenase 3
SPNS2	1.38	1.55	SPNS lysolipid transporter 2, sphingosine-1-phosphate
LIF	1.37	1.62	LIF interleukin 6 family cytokine
LONRF2	1.37	1.55	LON peptidase N-terminal domain and ring finger 2
GAL	1.35	1.56	galanin and GMAP prepropeptide
ERRFI1	1.34	1.51	ERBB receptor feedback inhibitor 1
EGR1	1.32	1.56	early growth response 1
JCAD	1.3	1.59	junctional cadherin 5 associated
APOBR	-1.08	-1.74	apolipoprotein B receptor
TPPP3	-1.1	-2.37	tubulin polymerization promoting protein family member 3
DBP	-1.13	-1.58	D-box binding PAR bZIP transcription factor
SYNDIG1	-1.28	-1.8	synapse differentiation inducing 1
SMIM39	-1.29	-1.86	small integral membrane protein 39
TMT1A	-1.39	-1.66	thiol methyltransferase 1A
PGM5	-1.48	-1.5	phosphoglucomutase 5
GPR68	-1.48	-1.87	G protein-coupled receptor 68
KIT	-1.51	-1.42	KIT proto-oncogene, receptor tyrosine kinase
MMP16	-1.53	-1.65	matrix metallopeptidase 16
SOX6	-1.63	-1.58	SRY-box transcription factor 6
ABCC9	-1.64	-1.9	ATP binding cassette subfamily C member 9
SEC14L5	-1.68	-1.94	SEC14 like lipid binding 5
MAP2K6	-1.8	-2.18	mitogen-activated protein kinase kinase 6
